# Clinical and Genetic Overview of Paroxysmal Movement Disorders and Episodic Ataxias

**DOI:** 10.3390/ijms21103603

**Published:** 2020-05-20

**Authors:** Giacomo Garone, Alessandro Capuano, Lorena Travaglini, Federica Graziola, Fabrizia Stregapede, Ginevra Zanni, Federico Vigevano, Enrico Bertini, Francesco Nicita

**Affiliations:** 1 University Hospital Pediatric Department, IRCCS Bambino Gesù Children’s Hospital, University of Rome Tor Vergata, 00165 Rome, Italy; giacomo.garone@opbg.net; 2 Movement Disorders Clinic, Neurology Unit, Department of Neuroscience and Neurorehabilitation, IRCCS Bambino Gesù Children’s Hospital, 00146 Rome, Italy; alessandro.capuano@opbg.net (A.C.); federica.graziola@opbg.net (F.G.); 3 Unit of Neuromuscular and Neurodegenerative Diseases, Department of Neuroscience and Neurorehabilitation, IRCCS Bambino Gesù Children’s Hospital, 00146 Rome, Italy; lorena.travaglini@opbg.net (L.T.); ginevra.zanni@opbg.net (G.Z.); enricosilvio.bertini@opbg.net (E.B.); 4 Laboratory of Molecular Medicine, IRCCS Bambino Gesù Children’s Hospital, 00146 Rome, Italy; fabrizia.stregapede@opbg.net; 5 Department of Neuroscience, University of Rome Tor Vergata, 00133 Rome, Italy; 6 Department of Sciences, University of Roma Tre, 00146 Rome, Italy; 7 Neurology Unit, Department of Neuroscience and Neurorehabilitation, IRCCS Bambino Gesù Children’s Hospital, 00165 Rome, Italy; federico.vigevano@opbg.net

**Keywords:** hyperkinetic movement disorders, dyskinesia, ataxia, cerebellum, basal ganglia, therapy, acetazolamide, epilepsy, whole exome sequencing, functional movement disorders

## Abstract

Paroxysmal movement disorders (PMDs) are rare neurological diseases typically manifesting with intermittent attacks of abnormal involuntary movements. Two main categories of PMDs are recognized based on the phenomenology: Paroxysmal dyskinesias (PxDs) are characterized by transient episodes hyperkinetic movement disorders, while attacks of cerebellar dysfunction are the hallmark of episodic ataxias (EAs). From an etiological point of view, both primary (genetic) and secondary (acquired) causes of PMDs are known. Recognition and diagnosis of PMDs is based on personal and familial medical history, physical examination, detailed reconstruction of ictal phenomenology, neuroimaging, and genetic analysis. Neurophysiological or laboratory tests are reserved for selected cases. Genetic knowledge of PMDs has been largely incremented by the advent of next generation sequencing (NGS) methodologies. The wide number of genes involved in the pathogenesis of PMDs reflects a high complexity of molecular bases of neurotransmission in cerebellar and basal ganglia circuits. In consideration of the broad genetic and phenotypic heterogeneity, a NGS approach by targeted panel for movement disorders, clinical or whole exome sequencing should be preferred, whenever possible, to a single gene approach, in order to increase diagnostic rate. This review is focused on clinical and genetic features of PMDs with the aim to (1) help clinicians to recognize, diagnose and treat patients with PMDs as well as to (2) provide an overview of genes and molecular mechanisms underlying these intriguing neurogenetic disorders.

## 1. Introduction on Paroxysmal Movement Disorders and Episodic Ataxias

**Figure 1:**
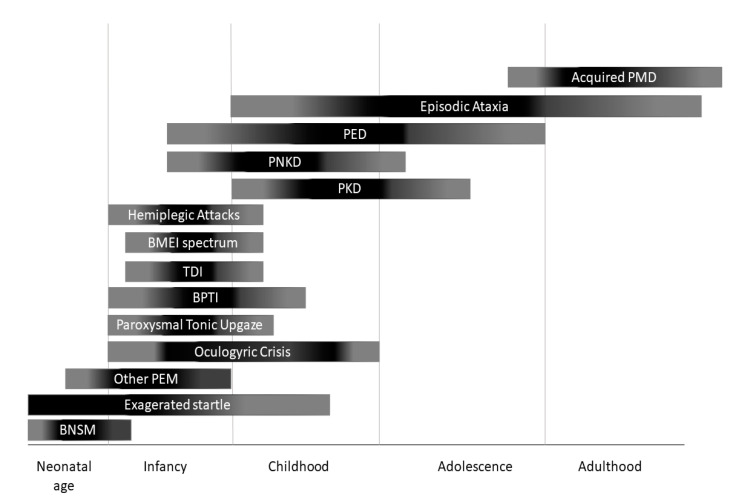
Onset of different paroxysmal movement disorders (PMDs) according with age. BNSM: benign neonatal sleep myoclonus; BMEI: benign myoclonus of early infancy; BPTI: Benign paroxysmal torticollis of infancy; PEM: Paroxysmal eye movements; PED: Paroxysmal exercise-induced dyskinesia; PKD: Paroxysmal kynesigenic dyskinesia; PNKD: Paroxysmal non-kynesigenic dyskinesia.

Paroxysmal movement disorders (PMDs) are rare neurological diseases typically manifesting with intermittent attacks of abnormal involuntary movements [1]. The term “paroxysmal” indicates a well-defined onset and termination of clinical manifestations. Two main categories of PMDs are recognized based on phenomenology: Paroxysmal dyskinesias (PxDs) are characterized by transient episodes hyperkinetic movement disorders, while attacks of cerebellar dysfunction are the hallmark of episodic ataxias (EAs) [2]. From an etiological point of view, both primary (genetic) and secondary (acquired) causes of PMDs are recognized. Some aspects of clinical history may help to distinguish primary from secondary PMDs: Most primary forms occur as sporadic or familial cases with autosomal dominant inheritance, and most often onset of manifestations is set in childhood or adolescence (Figure 1), and interictal neurological examination is unremarkable; secondary forms occur sporadically, more usually begin after the second decade of life (Figure 1), and clinical examination is frequently abnormal also outside of attacks. 

A further category that may manifest as PMDs are functional (psychogenic) movement disorders (FMDs). Patients with FMDs may show tremor, dystonia, myoclonus, parkinsonism, speech and gait disturbances, or other movement disorders whose patterns are usually incongruent with that observed in organic diseases, although sometimes diagnosis may be challenging. Diagnosis of FMDs is based on positive clinical features (e.g., variability, inconsistency, suggestibility, distractibility, and suppressibility) during physical examination and should be considered in presence of some clues such as intra-individual variability of phenomenology, duration and frequency of attacks, and/or precipitation of the disorder by physical or emotional life events. Other supporting information can be helpful (i.e., neurophysiologic and imaging studies) [3]. 

**Figure 2:**
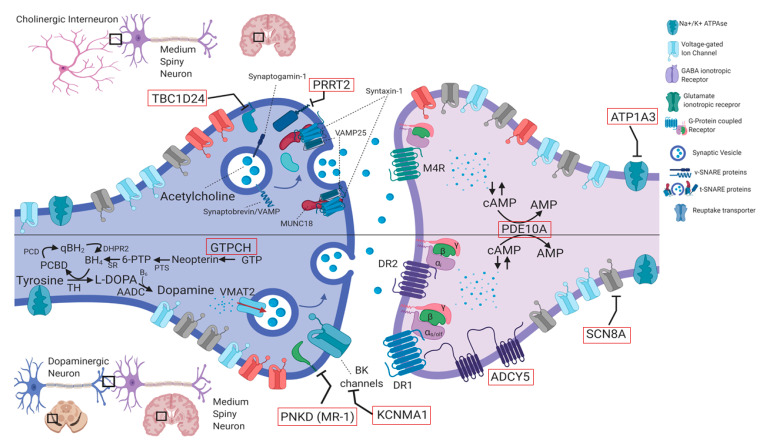
Schematic representation of synaptic neurotransmission mechanisms affected in PMDs in basal ganglia circuits. For simplicity, two hypothetical striatal synapses are shown: The synapse between a cholinergic interneuron and a medium spiny neuron (**top**), and the synapse between a dopaminergic neuron from the *substantia nigra pars compacta* and a striatal medium spiny neuron (**bottom**). Both these types of synapses are critical for control of volitional movements in humans. Red rectangles indicate genes involved in PMDs. Calcium channels are depicted in red, sodium channels in grey, potassium channels in blue. PMD: Paroxysmal movement disorders, GTPCH: GTP cyclohydrolase I, PTS: 6-Pyruvoyl Tetrahydrobiopterin Synthase, SR: sepiapterin reductase, BH4: Tetrahydrobiopterin; PCBD: pterin-4α-carbinolamine, PCD: pterin-4α-carbinolamine dehydratase, qBH2: quinonoid dihydrobiopterin; DHPR2: dihydropteridine reductase; TH: Tyrosine Hydroxylase; AADC: Aromatic l-amino acid decarboxylase, B6: pyridoxal phosphate (active form of vitamin B6); VMAT2: Vesicular monoamine transporter 2 (encoded by the *SLC18A2* gene), (c)AMP: (cyclic) adenosine monophosphate. DR1: dopamine receptor type 1; Dopamine receptor type 2.

**Figure 3:**
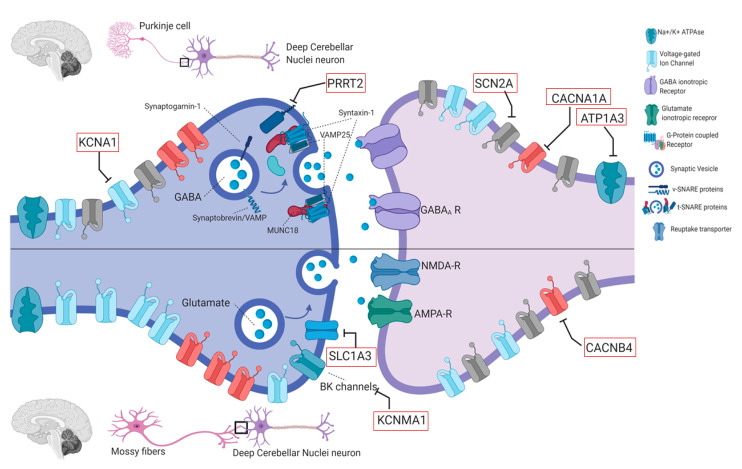
Schematic representation of synaptic neurotransmission mechanisms affected in PMDs in cerebellar circuits. For simplicity, two hypothetical synapses are shown: The GABAergic synapse between a Purkinje cell and a neuron of deep cerebellar nuclei (**top**), and the synapse between a glutamatergic cerebellar afferent (mossy fiber) and a neuron of deep cerebellar nuclei (**bottom**). Both these types of synapses are critical for cerebellar integration and coordination of movements. Red rectangles indicate genes involved in PMDs. Calcium channels are depicted in red, sodium channels in grey, potassium channels in blue.

**Figure 4:**
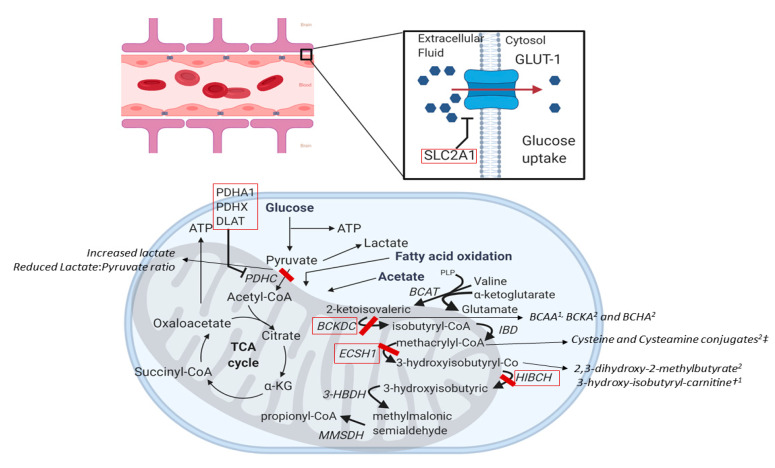
Molecular mechanisms causing brain energy failure and mitochondrial dysfunction in PMDs. Red rectangles indicate genes involved in PMDs. Expression and function of GLUT-1 on membrane surface of endothelial cells of the brain vasculature is illustrated on the top. Mitochondrial energy production and BCAA (leucine, isoleucine, and valine) catabolism are illustrated on the bottom. PDHC converts pyruvate into acetyl-CoA, regulating its entry into the tricarboxylic acid (TCA) cycle and the activity of the oxidative phosphorylation. PDHC deficiency decreases the availability of acetyl-CoA for the TCA cycle promoting the reduction of pyruvate to lactate, determining intracellular energy failure and impaired redox state. Metabolic defects in BCAA metabolism cause the production of toxic compounds, that alter mitochondrial function. In addition, in the central nervous system transamination of BCAA is a source of glutamate, that can be use as neurotransmitter or for further production of GABA. In ECSH1 and HIBCH deficiencies, the accumulation of methacrylyl-CoA and acryloyl-CoA and their sulphurated conjugates probably leads to secondary decreased activity of PDHC and mitochondrial respiratory chain complexes. In BCKD complex deficiency, the elevated leucine levels alter water homeostasis causing cerebral edema and dysmyelination and displace other essential amino acids impairing neurotransmission. In addition, α-ketoisocaproic acid (not shown), an intermediate in leucine metabolism, has toxic effects in the central nervous system. ‡ Acryloyl cysteine, Acryloyl N-acetylcysteine, Acryloyl cysteamine, Methacryl-cysteamine, Methacryl-l-cysteine, N-acetyl-acryloyl-cysteine. † The increase of 3-hydroxy-isobutyryl-carnitine distinguishes HIBCH from ECSH1 deficiency. ^1^ Detectable in plasma. ^2^ Detectable in urines A-KG: α-ketoglutarate; BCAA: branched-chain amino acids BCKA: branched-chain ketoacids; BCHA: branched chain hydroxyacids. BCAT: branched-chain amino acid aminotransferases; BCKDC: branched-chain α-keto acid dehydrogenase enzyme complex; IBD: Isobutyryl-CoA dehydrogenase; ECSH1: short-chain enoyl-CoA hydratase; HIBCH: 3-Hydroxyisobutyryl-CoA hydrolase; 3-HBDH: 3-Hydroxyisobutyrate-CoA dehydrogenase; MMSDH: Methylmalonic semialdehyde dehydrogenase. PDHC: Pyruvate dehydrogenase complex.

Recognition and diagnosis of PMDs are based on personal and familial medical history, physical examination, detailed reconstruction of ictal phenomenology (possibly including video-recording of at least one attack), brain magnetic resonance imaging (MRI), and genetic analysis. Neurophysiological (i.e., standard electroencephalogram or long-term monitoring) or laboratory tests are reserved for cases in which an epileptic origin of the attack cannot be excluded, or brain MRI reveals alterations that are compatible with genetic-metabolic or secondary causes. Genetic knowledge of PMDs has been largely incremented by the advent of next generation sequencing (NGS) methodologies, which allowed to increase both molecular diagnosis and identification of ultra-rare or new genes. The wide number of genes involved in the pathogenesis of PMDs (Table 1) reflects a high complexity of molecular bases of neurotransmission in cerebellar and basal ganglia circuits (Figure 2, Figure 3 and Figure 4). This comprehensive review is focused on clinical and genetic features of PMDs according to current nosology (Table 2). As this review is mainly targeted on genetic causes of PMDs, functional PMDs will not be discussed further. 

## 2. Methods

Bibliographic search has been performed on PubMed for papers published in English from 1940 up to February 2020, using the key terms “paroxysmal dyskinesias”, “paroxysmal dystonia”, “paroxysmal choreoathetosis” “episodic ataxia”, ”paroxysmal ataxia”, “paroxysmal non-epileptic event” “developmental movement disorders”, “hemiplegic attacks”, “paroxysmal ocular movements”, “alternating hemiplegia”, “hyperekplexia”, “startle”, “PRRT2”, “MR-1”, “CACNA1A”, “KCNA1” “SCL2A1”, “GLUT1”, “ADCY5”, “ATP1A3”, “TBC1D24”, “PDH”, “BCKD”, “gene”, “genetic”, “mutation”, and “phenotype-genotype” both individually and in combination. According with their relevance for the purpose of this review, both articles (e.g., case series, case reports, original research articles, and reviews) and book’s chapters were selected for the final reference list.

As further detailed below, nosology and classification of PMDs can be confusing. The term “paroxysmal” is rather unspecific and it has been used with different meanings in the literature. In movement disorders literature, PMDs and PxDs are usually used as synonims, excluding EAs from this definition. In child neurology literature, paroxysmal abnormal movements have been variably defined as paroxysmal non-epileptic events or transient benign PMDs [4,5], respectively highlighting the main differential diagnosis or the developmental outcome, rather than the phenomenology. In addition, in patients who present interictal movement disorders, no absolute criteria exist to properly differentiate true PMDs from a sudden, transient worsening of the ongoing symptoms. For practical use and for the purposes of this review, a PMD is defined by an ictal phenomenology that clearly differs from the interictal condition, a discrete rather than gradual temporal limits of the episode, and by attacks that recur in a similar fashion. Accepting this definition, exacerbations of preexisting dystonia and status dystonicus are not considered PMD, although they are sometimes referred as “paroxysmal” [6] and may have (in milder cases) spontaneous resolution. By contrast, attacks of chorea and/or dystonia in patients with neurodegenerative ataxias or hereditary spastic paraplegias, for istance, have been included among the PMDs [7,8,9]. For the different diagnostic and management implications and for historical reasons, other hyperkinetic movement disorders are not considered PMDs, although they have a limited time duration and their phenomenology is easily distinguishable from the baseline condition. This is the case of l-DOPA-induced dyskinesias, that are obviously excluded from PMDs because they are a common complication of Parkinson disease (limiting the diagnostic uncertainty), their onset and cessation is related to l-DOPA administration and their treatment is highly specific and is part of the global management of the underlying disease [10]. 

For this review of PMDs, we included both PxDs and EA, but also developmental PMDs and other paroxysmal abnormal movements typically occurring in genetic neurological diseases, in order to provide a comprehensive review of these phenomena in both children and adults.

## 3. Phenotypic Classification and Treatment Options 

### 3.1 Paroxysmal Dyskinesias and Other Paroxysmal Movement Disorders 

PxDs are a heterogenous group of movement disorders characterized by recurrent attacks of dystonia, chorea, athetosis, and ballismus, or any combination thereof [1,11]. Although the first description of a PxD case probably dated back to the end of the 19th century [12,13], the three main forms of PxDs were described and recognized as movement disorders different from epilepsy or neuromuscular disorders between 1940 and 1977 [14,15,16]. 

In 1995, moving from the analysis of 46 patients, Demirkiran and Jankovic proposed a phenotypic classification of PxDs purely based on the trigger factors of the episodes, that is still largely used [11,17]. This classification includes four types of PxDs: Paroxysmal kinesigenic dyskinesia (PKD), paroxysmal nonkinesigenic dyskinesia (PNKD), paroxysmal exertion-induced dyskinesia (PED), and paroxysmal hypnogenic dyskinesia (PHD). This latter form was proposed in 1981 [18], but it was later reported that an epileptic origin of attacks was identifiable in most cases [19]. As a consequence, PHD (at least in its original description) is no longer considered a form of PxDs, but rather a variant of nocturnal frontal lobe epilepsy [20]. This classification rejects the phenomenology of attacks as an informative feature because each entity may present with the same phenomenology, which in addition is often only presumed based on witnesses’ or patients’ descriptions [1,11]. Accordingly, the term dyskinesia was adopted for all the forms. Based on etiology, PxDs is further classified as primary (or idiopathic) and secondary [17].

Over the recent years, Demirkiran’s and Jankovic’s classification has been challenged with the latest genetic discoveries. It is now evident that most of the primary forms are rather secondary to a genetic defect and a marked genetic and phenotypic heterogeneity exists, a given PxD underlying different genetic defects and the same gene being involved in various PxDs [2]. As a result, different classification systems combining both phenotypic and genotypic information are emerging [1,21]. 

Despite genetic advances, the clinical classification still represents a pivotal framework for a correct interpretation of the attacks and to guide diagnostic workup, including genetic testing. Nevertheless, it should be considered that not all the PxD attacks fall in these entities, and that some patients may present more than one type of PxD [2,21].

#### 3.1.1 PKD (Paroxysmal Kinesigenic Dyskinesia)

The occurrence of sudden voluntary movements as the factor triggering paroxysmal attacks of dyskinesias was first described in 1967, when the term “paroxysmal kinesigenic choreoathetosis” was coined [15]. The diagnostic criteria currently adopted were defined in 2004 [22] and they include the occurrence of paroxysmal episodes of chorea and/or dystonia, the presence of a kinesigenic trigger, the short duration of episodes (less than 1 minute), an age of onset between 1 and 20 years, the absence of pain or consciousness impairment and response to antiepileptic drugs (AEDs) modulating voltage-gated sodium channels (phenytoin or carbamazepine). 

The attacks can involve the limbs, trunk and face in a focal, multifocal or generalized manner. The frequency of attacks can be very high (up to 100 per day), but it tends to decline after puberty, with remission in up to 50% of the cases [11,23]. The trigger is usually represented by gross movements or acceleration of ongoing movements. Several patients report that a sensory aura or a premonitory sensation could precede the attacks [22]. 

Defects in the *PRRT2* gene were the first cause of PKD to be identified [24], accounting for 27%–65% of cases in different cohorts [25,26,27,28,29,30,31,32]. Other genes have been implicated in the pathogenesis of PKD, including *SCN8A*, *SLC2A1*, *DEPDC5*, *PNKD*, *KCNMA1*, *KCNA1*, *CHRNA4*, and *SLC16A2* [21,33,34,35,36,37,38]. Unusually, PKD has been reported in various acquired or neurodegenerative conditions [8,39,40,41,42,43,44].

#### 3.1.2. PNKD (Paroxysmal Nonkinesigenic Dyskinesia)

First described by Mount and Reback in 1940 [14], the phenotype of PNKD is more variable than PKD and is rapidly expanding [20]. Classic PNKD paroxysms show a combination of dystonia, chorea, and athetosis that involve one side and subsequently spread to the other side, involving the limbs and the face. The attacks typically last from 10 minutes to 1 hour, but up to 4 hours or even an entire day, and occur with low frequency, few times per weeks or just with few episodes in a lifetime. Consciousness is preserved, but bulbar involvement may prevent speaking [11]. Trigger factors include fatigue, emotional stress, and consumption of caffeine, tobacco, or alcohol. A premonitory sensation is frequently reported, namely limb numbness, stiffness or restlessness sensation, but prodromal symptoms may involve also headache and hyperventilation [11,45]. In most cases, sleep relieves the paroxysms [45,46,47]. 

In typical cases, the onset is in infancy or childhood and the interictal neurological examination is normal [20,22]. Familial cases show an autosomal dominant pattern of inheritance with high penetrance [23,48]. Atypical PNKD attacks may feature blepharospasm, *risus sardonicus*, and laryngeal dystonia [45,49], or variate in frequency or duration [23]. 

The cornerstone of treatment is avoidance of precipitating factors, while benzodiazepines are used to prevent or relieve the attacks [23]. In some cases, response to different AEDs (such as levetiracetam, valproic acid, acetazolamide, gabapentin, or oxcarbazepine) [50,51,52] or deep brain stimulation [53] has been reported.

Classic, isolated forms are mainly due to mutations in the PNKD gene, formerly known as MR-1 [21,23]. Paroxysmal attacks of dystonia and/or chorea with heterogenous phenomenology have been reported in several other genetic diseases, including PRRT2, SLC2A1, ATP1A3, ADCY5, TBC1D24, KCNMA1, PDE10A, and KCNA1-related conditions, usually as part of complex phenotypes and lacking typical triggers [21,54,55]. In addition, many acquired PxDs manifest as PNKD [20,22,56].

#### 3.1.3. PED (Paroxysmal Exercise-Induced Dyskinesia)

The occurrence of paroxysmal episodes of dystonia and chorea after prolonged exercise was first described in three members from a single family in 1977 [16]. Paroxysms usually last 5–30 minutes (rarely up to 2 hours) and usually affect the body part involved in the sustained exercise (frequently the legs). Attacks can be also triggered by fasting, stress, sleep deprivation or, more rarely, by cold, muscle vibration, or passive movements, and are usually relieved by eating or resting [11,23]. Frequency of the paroxysms depends upon the level of physical exercise and the exposure to other precipitating factors, varying between daily attacks to few episodes per month [11]. Onset is usually during infancy or childhood but can be as late as in young adulthood [11].

PED are genetically heterogenous, but mutations in the *SLC2A1* gene are the most common causative defect, presenting as the only manifestation or as part of complex phenotypes [1,57,58]. PED can also reflect other defects in brain energy and mitochondrial metabolism, dopaminergic deficiency, or neurodegenerative conditions [59,60,61,62,63,64,65,66,67]. Effective treatment depends upon the involved molecular mechanism.

#### 3.1.4. Paroxysmal Nocturnal Dyskinesia 

Although the originally described form of PHD has been reclassified as a variant of frontal lobe epilepsy in which the epileptic focus can be difficult to detect [19,20], the occurrence of nocturnal bouts of non-epileptic hyperkinetic movements has been described in different conditions, questioning the exclusion of PHD from PxDs [21]. 

Nocturnal paroxysmal movements are a core feature of *ADCY5*-related movement disorder, as part of a complex and pleiotropic motor phenotype including both paroxysmal and ongoing abnormal movements [68,69]. Nocturnal, non-epileptic, short (<1 min) attacks of chorea and ballism occurring in NREM sleep have also been reported in patients carrying *PRRT2* mutations [70]. In addition, paroxysmal nocturnal dyskinesias have been reported in various acquired conditions [71,72].

#### 3.1.5. Developmental Paroxysmal Movement Disorders

Paroxysmal motor phenomena are quite frequent in children and many of them have a transient and benign course, with complete remission without neurological or developmental sequelae [73]. For this reason, a group of movement disorders appearing between neonatal period to early childhood is variably described under the umbrella terms of “developmental and benign movement disorders”, “transient benign paroxysmal movement disorders”, or “paroxysmal non-epileptic motor events” [4,5,73]. Although these entities are usually described as transient phenomena occurring in otherwise typically developing children, more rarely they could represent early features of genetic disorders or underlie structural brain lesions [74,75,76]. In these cases, neurological abnormalities and developmental delay usually coexist.

*Benign neonatal sleep myoclonus* presents in newborns with paroxysmal jerks that occur only during sleep (almost exclusively in quiet sleep) and cease immediately on arousal [73]. The myoclonic jerks may be focal or multifocal, uni- or bilateral (sometimes with migrating appearance), synchronous or asynchronous, rhythmical or arhythmical. Myoclonic bouts last several minutes (up to 90 minutes) [5]. Frequency of attacks usually decreases by the second month of life, with full remission before 6 months in most of the cases [77]. Ictal and interictal EEG, as well as developmental trajectory and neurological examination are normal. The anatomical origin of the jerks is unclear, but a spinal generator has been proposed [78]. 

*Paroxysmal tonic upgaze* (PTU) appears in early infancy with paroxysms of conjugate upward deviation of the eyes, associated with down-beating saccades on attempts to downward gaze, preserved horizontal eye movements and unimpaired consciousness [5,74]. During attacks, the neck is usually flexed in a chin-down position, apparently in the effort to compensate the involuntary abnormal position of the eyes [5]. Eyes deviation can be sustained or intermittent, with clusters of recurrent attacks lasting 2–30 s [73]. The entire episode lasts hours (but up to 48 h in rare cases) and variable degrees of ataxia may be associated [79]. Frequency of attacks ranges from 2 to 10 attacks/day, and they usually gradually resolve by the age of 4 years [79,80]. Despite the remission of the attacks, persistence of neurological abnormalities such as ataxia, subtle oculomotor abnormalities and developmental and/or language delay in children with PTU has been documented [73]. The physiopathology of PTU is unclear: A possible dystonic nature has been claimed, as well as an age-related defect in mesencephalic supranuclear control of vertical gaze or cerebellar integration [5,73,74]. In cryptogenic cases, neurophysiological, laboratory, and neuroimaging investigations are normal. Genetic studies have shown that PTU is a common early feature of *CACNA1A*-related disease [74,81,82,83]. In addition, PTU has been documented in patients with *GRID2* mutations [84] and in association with genetic or acquired structural brain lesions, including white-matter disease, perinatal injury, hydrocephalus, brain tumors, or brain malformations [5,73]. In a few cases, response to l-DOPA has been reported [79,85], raising the issue of distinction between PTU and oculogyric crisis (OGC), observed in biogenic amines synthesis defects [73]. In other cases, carbonic anhydrase inhibitors proved to be effective [74,81]. In conclusion, the exact nosological boundaries and etiology of PTU remain to be elucidated.

*Benign paroxysmal torticollis* (BPT) presents as recurrent episodes of abnormal, painless head postures, alternating from side to side [5]. It consists of paroxysmal cervical dystonia featuring latero-, retro-, or torticollis, with occasional trunk involvement (tortipelvis). Attacks may last from few minutes to several days. Onset is usually before 3 months of age but may range from the first weeks of life to 30 months [73]. Autonomic signs (pallor, nausea, vomiting) and ataxia may coexist and also become the prominent features over time [86]. Usually, BPT resolves by 5 years of age [5]. Frequent later appearance of migraine has been widely reported [86,87,88], suggesting that BPT should be considered an age-dependent migraine disorder to include among periodic syndromes of childhood [5]. The evidence that *CACNA1A* mutations segregated in several families showing variable combinations of BPT, PTU, EA, hemiplegic migraine, and benign paroxysmal vertigo further supports the hypothesis of a common pathophysiological background for these phenomena [75,82,83,89,90]. Treatment is not usually needed, unless irritability, discomfort, or vomiting necessitate symptomatic management. The study of developmental trajectories of children with BPT showed conflicting results [89,91,92], suggesting that careful neurodevelopmental follow-up is needed.

*Transient dystonia of infancy* consists of paroxysmal episodes of abnormal upper limb posture, with occasional concomitant involvement of the trunk a single lower limb [73]. The dystonic posture usually resolves with volitional movements. Interictal examination and neuroimaging are normal. The age of onset is usually between 5 and 10 months, progress for a time and gradually resolves in 3 months to 5 years, without developmental or neurological sequelae [93,94,95]. Its etiology and pathophysiology are unclear. 

*Benign myoclonus of early infancy* (BMEI) was originally described as a non-epileptic paroxysmal motor disorder characterized by the occurrence of myoclonic jerks of the head and/or of the upper limbs, usually occurring in clusters, mimicking infantile spasms [96]. Instead, shuddering attacks (SA) were defined as brief (5–15 s) paroxysmal bursts of head and/or shoulders tremor [97]. In both cases, consciousness is preserved, and attacks usually occur during wakefulness, more rarely in sleep or drowsiness. Ictal EEG, neurological status and development need to be normal to confirm the diagnosis [73]. To date, both BMEI and SA are considered as part of the same spectrum of PMDs, sometimes defined as benign polymorphous movement disorder of infancy [5]. Beside myoclonic jerks and SA, the spectrum of possible manifestations broadened to include spasms and brief tonic contractions [98], atonic and negative myoclonus (usually head drops) [98,99] and diffuse movements (“shaking body attacks”) [100], with possible coexistence of different episodes in the same patient, simultaneously or sequentially. The attacks usually have abrupt onset, and frequently appear in clusters. Each attack usually lasts few seconds, but multiple episodes per day often occur, frequently triggered by excitement, frustration, postural changes, or sensory stimuli [5]. Onset is in the first year of life (mainly between 4 and 7 months), the attacks usually cease by the age of 2 years but sometimes persist into childhood. Pathophysiology is unknown and no treatment is needed, except for parents’ reassurance.

#### 3.1.6. Other Paroxysmal Movement Disorders in Pediatric Neurological Diseases

Besides PxDs and developmental PMDs, various paroxysmal motor disorders have been reported in different neurological diseases with pediatric onset. 

Exaggerated startle response is a feature of different neurologic conditions [101]. *Hereditary hyperekplexia* is a neurogenetic disease with neonatal onset, manifesting with the classic triad of generalized stiffness after birth (resolving during the first years of life), excessive startling to unexpected auditory or tactile stimuli (which persist all lifelong), and generalized stiffness after the startle reflex, lasting few seconds [102]. In newborns, startle reflex may elicit violent jerks, followed by sustained stiffening of trunk and limbs, with high-frequency trembling [103]. Minor forms presenting only with excessive startle response and onset in infancy or childhood may occur [102]. To date, four genes responsible for hereditary hyperekplexia have been identified (*GLRA1*, *GLRB*, *SLC6A5*, and *ATAD1*), with *GLRA1* accounting for most of the cases [104]. Exaggerated startle reflex is also an early, typical feature of GM1 gangliosidosis, usually identifiable within the first 2 months of life [105]. In addition, “symptomatic” hyperekplexia has been described in a broad range of genetic and acquired encephalopathies [105,106].

*Hemiplegic attacks* (HA) involving either one side of the body, alternating in laterality, are the hallmark of alternating hemiplegia of childhood (AHC), due to *ATP1A3* mutations. In AHC, HA present within the first 18 months of life [107]. In addition, HA have been associated with mutations in *ATP1A2*, *SLC2A1*, *SCN4A*, *ADCY5*, *TBC1D24*, and *TANGO2* genes [108,109,110,111,112,113]. Few patients presenting HA exclusively out of sleep, with no appearance of other PMD or progression to neurologic and intellectual involvement, have been reported. This mild disorder is considered a separate entity known as benign nocturnal alternating hemiplegia of childhood [114]. The genetic cause (if any) of this form is unknown. 

*Paroxysmal ocular movements* are another possible (and often early) feature of several neurological diseases. OGC consist of paroxysmal, tonic, conjugate, often upward ocular deviation lasting minutes to hours. Their phenomenology is indistinguishable from PTU. OGC are considered a dystonic manifestation resulting from dopaminergic dysfunction, reported in primary disorders of biogenic amine synthesis, iatrogenic dopamine receptor blockade, brainstem encephalitis or neurodegenerative syndromes affecting dopaminergic pathways [115]. Paroxysmal brief episodes of eye–head movements (“saccadic eye-head gaze shifts”) have also been reported as early manifestations of GLUT-1 deficiency syndrome (GLUT1-DS) due to *SLC2A1* mutations [116,117]. In addition, paroxysmal ocular movements (monocular and binocular nystagmus, dysconjugate gaze, ocular bobbing, ocular flutter) are often the first neurological manifestation of AHC, preceding the onset of HA [118].

### 3.2. Episodic Ataxias 

EAs are clinically and genetic heterogeneous disorders characterized by recurrent and paroxysmal cerebellar dysfunction presenting as attacks of ataxia and poor coordination with a well-distinct onset and termination of clinical manifestations [119,120]. Together with the more common autosomal dominant spinocerebellar ataxias (SCAs), they belong to the group of the autosomal dominant cerebellar ataxias. EAs are rare neurogenetic disorders: The overall estimated incidence of EAs is less than 1/100.000 subjects [121]. Duration of a single attack can range from a few seconds to hours or days. Consciousness is preserved during the attack and, beyond cerebellar ataxia, a combination of additional ictal features such as dysarthria, tremor, vertigo, nausea, diplopia, dystonia, hemiplegia, headache, and tinnitus may be present. Neurological interictal examination may be completely normal or reveal the presence of persistent cerebellar ataxia (sometimes progressive, thus making difficulty in distinguishing EA with progressive features from progressive ataxia with intermittent exacerbation), and a variable combination of myokymia, extrapyramidal signs, epilepsy or cognitive impairment [119,120]. EAs usually start in the first two decades of life—mainly during childhood or adolescence—and recurrence of ataxic attacks is variable, ranging from a few episodes per year to several per day. Attacks may be triggered by several factors such as drinks (i.e., alcohol, caffeine), systemic conditions (i.e., fever, fatigue, psychological stress) or other (i.e., sudden movements, startle, emotional stimuli) [119,120,121]. Currently eight different forms of sporadic or familial autosomal dominant EAs are classified in the Online Mendelian Inheritance in Men (OMIM) classification system, although causative genes are known for five of these eight subtypes. However, with the exception of EA1 and EA2, which represent the majority of EAs patients and show recognizable ictal and interictal phenotypes, most of the remaining EAs are very similar to each other. 

Additionally, in the last few years mutations in novel genes or in genes previously associated with other neurogenetic disorders have been discovered in EAs patients, although these forms have still not been classified in OMIM. Finally, EA may be the presenting symptom of some genetic-metabolic diseases or also acquired disorders of the CNS. With regards to treatment of EAs, specific classes of drugs are available, although so far no trials comparing efficacy has been completed and no drug has been definitively proven to be effective. The EAT2TREAT study, which compares sustained-release form of 4-amynopiridine versus acetazolamide versus placebo is currently ongoing. Management of EAs is based on the use of sodium channel blockers AEDs—such as phenytoin, carbamazepine and lamotrigine—or of carbonic anhydrase inhibitors—such as acetazolamide [122]. Sodium channel blockers may act by stabilizing the inactivated state of voltage-gated Na^+^ channels, with subsequent reduction of availability to channel’s opening [123]. Mechanisms of action of acetazolamide in EAs are speculative: It is believed to act by modulating bicarbonate ions gradient or intracellular pH—with subsequent modulation of neuronal membranes hyperpolarization with decreased excitability [123]. Long-term use of phenytoin should be carefully considered in EAs patients since it has been related with cerebellar dysfunction and atrophy [124]. Long-term side effects of acetazolamide are nephrolithiasis, hyperhidrosis, paresthesias of the extremities, rash, weakness, and gastrointestinal problems; this drug should be avoided in patients with liver, renal, or adrenal insufficiency [123]. Patients who develop persistent cerebellar syndrome in the context of EAs will benefit from physiotherapy, speech, and occupational therapies. 

#### 3.2.1. EA1

EA type 1 has been firstly described in 1975 in a three-generation family with autosomal dominant transmission of early childhood, short-lasting episodes of ataxia followed by onset of myokymia of the face and limbs in the second decade of life [125]. Myokymia is an involuntary muscular contraction manifesting with undulating, vermicular, rippling, or wavelike movement spreading across the muscle surface. Myokymia may be focal, most commonly involving the facial muscles, or may be generalized with involvement of extremities. Myokymia may be clinically visible or detected by electromyography, which reveals regular groups of motor unit discharges, especially doublets and triplets, occurring with a regular rhythmic discharge and distinguishing it from fasciculations. Several sporadic or familial cases have been reported and the natural history has been characterized [126]. EA1 is caused by heterozygous mutations in *KCNA1* although some individuals with typical EA1 symptoms have been reported not to carry *KCNA1* variations, suggesting that other unidentified genes may underlie the disorder.

EA1 begins in childhood and in almost all cases before the age of 20 with episodes of cerebellar ataxia and vertigo lasting less than 15 minutes; persistent myokymia is present between attacks [126]. The episodes of ataxia can appear spontaneously or can be triggered by physical activity/exercise, stress, environmental temperature, fever, caffeine or alcohol, and pregnancy or menstruation in women [126]. A variable combination of other signs and symptoms such as dysarthria, tremors, diplopia, blurred vision, vertigo, stiffening of the body, nausea, migraine, or diaphoresis may also be present during attacks. Severity of attacks is variable: some patients may be able to walk independently while others may require a support or may not be able to walk. The frequency of the ataxic episodes is variable even in patients with the same genotype (e.g., *KCNA1* variants, see later): Some patients experience several episodes per day, while others less than one per month. Number of attacks generally decreases with age, suggesting that some compensatory mechanisms may take place in the brain [127]. Interictal neurological examination and history are usually unrevealing, although other features such as delayed motor development, cognitive disability, choreo-athetosis, neuromyotonia, or progressive cerebellar ataxia have been reported [119,120,121,126]. In particular, the presence of permanent cerebellar signs has been observed in 20% of large series and it is related to disease duration [126]. Co-existence of epilepsy is reported in 10% of EA1 patients [128]. Brain MRI is usually normal but may reveal (10%) cerebellar atrophy [126]. Atypical phenotypes of EA1 may comprise absence of myokymia [129], paroxysmal dyspnea [130], isolated severe neuromyotonia [131], association with malignant hyperthermia susceptibility [132], isolated cataplexy triggered by sudden physical exertion [133], muscle spasms and rigidity [133], or intermittent myokymia tremor-like (without ataxia), which may be misdiagnosed as essential tremor or PKD [134]. Pharmacological therapy of EA1 is based on the use of carbamazepine. Occasional response has been reported also under phenytoin and acetazolamide treatment. It is worth pointing out that the therapeutic response is variable even among individuals with the same genotype. 

#### 3.2.2. EA2

EA2 is the most common form of EA, with an estimated prevalence of 1/100.000 [119,120,121]. Onset is usually placed in childhood or early adolescence (age range 2–32 years) although late-onset cases (i.e., in the fifth or sixth decade) have been reported. Ataxic episodes are triggered by physical stress, exercise or drinks (i.e., alcohol and coffee) and last up to several hours or days. Patients experience trunk and limb ataxia randomly associated with dizziness, dysarthria, migraine, nausea and vomiting, diplopia, tinnitus, and generalized muscle weakness. As for EA1, an epilepsy syndrome is a frequent finding in EA2 patients [135]. Before the onset of EA some patients may have experienced other PMDs during infancy or childhood such as PTU or paroxysmal torticollis [74,82,83,136]. Interictal examination is normal or may reveal permanent cerebellar ataxia and different subtypes of nystagmus (i.e., gaze-evoked, rebound or primary position downbeat nystagmus) [137,138,139]. Frequency and severity of episodes as well as associated signs and course of disease is highly variable among EA2 patients, also in the same family. There are more severe forms that manifest with hypotonia in the first months of life, followed by a delay in acquiring walking and learning difficulties [74,136,140,141]; in patients with late onset, the manifestations observed are often dizziness, instability and permanent balance disorders. On brain imaging, cerebellar atrophy with vermis predominance can be observed. EA2 is caused by heterozygous variants in the calcium channel, voltage-dependent, P/Q type, and α1A subunit (*CACNA1A*) gene [136]. Acetazolamide is the drug of choice for reducing ataxic attacks in EA2 [122]. Starting dose is usually of 250 mg/day divided in two doses, although effective daily dosage may range between 250 and 1000 mg [122]. Efficacy of 4-aminopyridine (at a dosage of 5 mg three times daily), a non-selective voltage-gated potassium channels blocker, in reducing frequency of attacks has been demonstrated in a randomized, double-blind, and placebo-controlled trial conducted on adolescent and adult patients with EA2 [142]. 4-aminopyridine may be used in EA2 patients who experienced acetazolamide-related side effects with subsequent discontinuation. Finally, an anecdotal case showed good response to combination of levetiracetam 750 mg/day and acetazolamide [143]. 

#### 3.2.3. EA5

EA5 has been firstly described in 2000 [144] and recently a further family has been reported [145]. The first description also included a family with epilepsy and without ataxia [144]. Main features of attacks in EA5 are similar to those observed in EA2 except for a later onset of manifestations in EA5. Long-term follow-up in one adult EA5 case has shown permanent ataxia. Available brain MRI data are consistent with normal findings. Positive response to acetazolamide has been reported. This subtype of EA is caused by heterozygous variants in the β4 subunit of the voltage-dependent calcium channel (*CACNB4*) [144]. 

#### 3.2.4. EA6

The first description of EA6 dates back to 2005, when Jen and colleagues [146] described a 10-year-old boy with a combination of EA, HA and epilepsy. Episodes of ataxia appeared first, from the age of 6 months, triggered by febrile illness. Later, at the age of six he developed alternating hemiplegia. Brain MRI discovered cerebellar atrophy and neurologic examination showed mild interictal truncal ataxia. An approach through a candidate gene allowed the identification of a de novo variant in the *SLC1A3* gene, also known as excitatory amino acid transporter 1 (*EAAT1*). The second described Dutch family had interictal nystagmus, milder episodes of acute ataxia lasting several hours, with onset in the first or second decade, response to acetazolamide, no seizures or alternating hemiplegia and resembled EA2 [147]. Phenotypical spectrum of EA6 has been confirmed [148] and successively expanded in additional descriptions of patients with adult-onset (e.g., third decade) fixed cerebellar ataxia without episodic features [149], late-onset (e.g., fifth and sixth decade) EA [150,151] and adult-onset EA with interictal nystagmus and intellectual disability [151].

#### 3.2.5. EA8 

The term EA8 was proposed to describe a large non-consanguineous Irish family in which affected members manifested episodes of unsteady gait and generalized weakness with dysarthria, triggered by physical fatigue or stress [152]. The symptoms of EA appeared in the second year of life as the children learn to walk. Additional features may include twitching around the eyes, nystagmus, myokymia, mild dysarthria, and persistent intention tremor. Frequency and duration of attacks were variable within the family. None of the affected members had epilepsy or tinnitus, but migraine with aura was recorded in two cases. Acetazolamide was ineffective in all cases, but interestingly episodes of ataxia responded to treatment with clonazepam. Whole exome sequencing led to the identification of two heterozygous variants in two different genes, *HSPG2* and *UBR4* genes, of which the latter was hypothesized to be the main candidate gene by the authors [152]. Recently, other patients with *URB4* mutations have been reported [151]. In their paper exploring the utility of whole exome sequencing in EAs, Choi and colleagues reported four patients with *URB4* mutations. Two patients had mutations in both *URB4* and *CACNA1A*, suggesting that *URB4* as a possible genetic modifier for EA2. The remaining two patients had onset of EA between the fourth and fifth decade, and interictal nystagmus was observed in one of them [151].

#### 3.2.6. Other EAs with Associated Disease Loci 

Other types of EAs have only been reported in few patients and families. The absence of pathogenic mutations in EA1 and EA2-related genes and some clinical features may help to distinguish these forms. 

*EA3* has been described in a large Canadian family with an autosomal dominant disorder characterized by persistent interictal myokymia and episodes of ataxia with dizziness, tinnitus, visual disturbances, and headaches lasting less then 30 minutes [153]. Attacks were controlled by acetazolamide. This form of EA differed from EA1 by the presence of ictal vertigo and tinnitus and from EA2 by the absence of interictal nystagmus. Furthermore, the variable age of onset observed in the family may help to distinguish this form from the typical EA1 (whose onset is in late childhood or early adolescence) as well as typical EA2 (whose onset is in childhood). Regarding the nomenclature, this form was initially described as EA4 and few years later as EA3 [154]. The disease locus was located in 1q42, but no gene was identified [154].

*EA4*, also known as *periodic vestibulocerebellar ataxia (PATX) or North Carolina autosomal dominant ataxia*, is an autosomal dominant disorder described in two families from North Carolina [155,156] and characterized by recurrent episodes of vertigo and ataxia associated with ocular abnormalities (diplopia, deficient smooth pursuit, gaze-evoked nystagmus). Attacks typically last for hours and acetazolamide is not effective. The age of onset varies from the third to sixth decades. Slowly progressive cerebellar ataxia has been recorded in some affected individuals. The EA1 and EA2 locus have been excluded, as well as SCA1–5 and dentatorubral-pallidoluysian atrophy but the gene is not identified [157]. However, neither the genetic locus nor the molecular defect causing EA4 have been identified [157]. Recently, a neuropathological study on a 91-year-old Caucasian female from a large family in North Carolina with EA4 was described [158]. The authors found clinical neuropathological similarities between EA4 and SCA type 6 (i.e., adult-onset and slowly progressive cerebellar ataxia with vestibulo-ocular abnormalities, cerebellar atrophy with distortion and loss of Purkinje cells, unremarkable brain stem, mild to moderate neuronal loss, cytoplasmic protein aggregates in Purkinje cells and neurons of the cerebellar dentate nucleus but an absence of ubiquitinated neuronal intranuclear inclusions) and hypothesized that CAG repeats may contribute to neuronal pathology observed in EA4, although it does not necessarily imply that EA4 is a triplet CAG repeat disorder [158]. 

*EA7* has been reported in one autosomal dominant family in which affected members presented before the age of 20 years with EA, weakness and slurred speech [159]. Episodes were triggered by exercise and stress and may last hours to days. Frequency ranged to monthly to yearly and it showed decrease with age. Overall, clinical features resemble EA2 but no nystagmus or additional interictal features were recorded on neurological examination. Gene locus was mapped at 19q13, but no causative gene was not identified.

## 4. Genetic Aspects and Pathophysiology

The genetic pleiotropy of PMD and EAs, leading to coalescing phenotypes, suggests that different genetic defects could converge into a limited range of disease mechanisms, resulting in similar manifestations. Most genes causing PMD and EAs encode for proteins involved in synaptic vesicle fusion (*PRRT2*, *PNKD*, *TBC1D24*), post-synaptic intracellular signaling (*ADCY5*), brain energy metabolism (*DLAT*, *PDHA1*, *PDHX*, *ECSH1*, *HIBCH*), neurotransmitter synthesis (*GCH1*), ion channels (*SCN8A*, *KCNMA1*, *ATP1A3*, *KCNA1*, *CACNA1A*, *CACNB4*, *SCN2A*) or solute carriers (*SLC2A1*, *SLC16A2*, *SLC1A3*) [21]. Disruption of synaptic neurotransmission, mostly affecting cerebellar and striatal circuitries, emerges as a common final effect (Figure 2 and Figure 3) [160]. It may variably result from dysfunctions in pre-synaptic fusion machinery, post-synaptic signaling pathways, neurotransmitters synthesis, reuptake and transport, neuron membrane excitability or brain energy production. With the notable exception of brain energy failure, the pathophysiological reason for the paroxysmal character of these disorders remains elusive, as well as the age-dependency of most of these manifestations.

### 4.1 Genes in Paroxysmal Movement Disorders

#### 4.1.1. PRRT2 (OMIM #614386)

*PRRT2* mutations were first linked to PKD in 2011 [24], and this gene was later found to cause also benign familial infantile seizures (BFIS) and infantile convulsions with choreoathetosis (ICCA) syndrome [1], leading to consider *PRRT2*-related paroxysmal disorders as a continuous phenotypic spectrum [161]. In addition, *PRRT2* has been linked to other paroxysmal neurological disorders, including PHD [70], EA (see later) [58] and hemiplegic migraine [161].

In 99% of *PRRT2*-PKD cases, a single heterozygous mutation is found. In less than 1% of the cases, a recurrent deletion of the 16p11.2 region including *PRRT2* could be the causative defect, usually with a more complex phenotype featuring developmental delay, intellectual disability, and/or autistic traits [162,163,164]. Few cases carrying biallelic mutations have been described, showing a more severe phenotype with various combinations of PxDs, EA, epilepsy and intellectual disability [165,166]. 

Compared to non-*PRRT2*-related PKD, in *PRRT2*-mutation carriers the age at onset is earlier, the episodes last longer and occur with higher frequency, phenomenology of attacks more frequently combines both dystonia and chorea and lower carbamazepine doses (50–100 mg/day) are effective [23,32,167,168]. 

About 70 different mutations have been reported so far, with no clear genotype–phenotype correlation and a marked phenotypic variability, also in the same family. The c.649dup p.(R217Pfs*8) is the most common variant [161]. Most mutations fall in the coding regions, but some splice-sites and intronic variants have been reported [161]. In classic, heterozygous cases, the penetrance of the disorder is incomplete (60%–90%) [169].

The proline-rich transmembrane protein 2 (PRRT2) encoded by the *PRRT2* gene is a 340-amino acid protein highly expressed in cerebral cortex, basal ganglia and cerebellum in the adult brain [170,171]. PRRT2 is neuron-specific, and is primarily located at a pre-synaptic level (mostly in axons), where it regulates neurotransmitter release by interaction with the synaptic vesicle fusion machinery [172]. Specifically, PRRT2 negatively regulates synaptic fusion modulating the vesicle priming process through interference with the SNARE complex assembly (Figure 2) [173]. Other possible mechanisms include a role in modulating the endocytic pathway, with regulation of the pool of neurotransmitter vesicles [174]. 

Most *PRRT2* frameshift mutations (including the c.649dupC mutation) affect messenger RNA stability or generate a truncated protein undergoing rapid degradation, suggesting a loss-of-function (LOF) mechanism [161]. This hypothesis is further supported by the *PRRT2*-knock-out mouse model, which closely replicates the paroxysmal disorder observed in humans [175]. Nevertheless, a dominant-negative effect for some missense variants is possible [161]. The age-dependent manifestation of *PRRT2*-related paroxysmal disorders (with BFIS occurring in children and resolving within early childhood and PKD beginning in childhood and remitting in adulthood) is not clear. It could reflect an age-related pattern of PRRT2 expression in different brain regions, with a temporal shift from cortex to basal ganglia during development [161].

#### 4.1.2. PNKD (Formerly MR-1) (OMIM #609023) 

Heterozygous *PNKD* mutations were first discovered as the causative defect in PNKD patients in 2004 [176]. The phenotype is rather homogenous with pure forms of PNKD, and the disorder is inherited with near-complete penetrance [23]. To date, three missense mutations have been found to cause PNKD (p.A7V, p.A9V, p.A33P), while a single-base deletion (p.P341X) has been reported to cause hemiplegic migraine in a single family. Rarely, *PNKD* mutations have been reported to cause PKD [34,58]. Intragenic or whole-gene deletions have not been reported so far. *PNKD* encodes for a 385-amino acid protein, whose isoform 1 is a membrane-associated protein selectively expressed in neurons and located at presynaptic terminals [177]. It inhibits neurotransmitter release through interaction with Rab3-interacting molecules, a family of pre-synaptic proteins involved in exocytosis facilitation [178]. In murine models, *PNKD* mutations act with a gain-of-function (GOF) effect, disrupting nigrostriatal neurotransmission and causing abnormal dopamine release in response to caffeine and ethanol [177].

#### 4.1.3. SLC2A1 (OMIM #138140) 

*SLC2A1* encodes for the GLUT-1, the primary glucose carrier in placenta, erythrocytes, and brain. GLUT-1 is highly expressed on brain endothelial cells, where it mediates glucose transport across the blood–brain barrier (BBB, Figure 4) [179,180]. *SLC2A1* haploinsufficiency is responsible for a wide spectrum of neurological abnormalities, ranging from classic GLUT1-DS (a severe, infantile-onset encephalopathy causing epilepsy, microcephaly, developmental delay and complex movement disorders) to milder forms, showing variable combinations of cognitive disabilities, different seizure types, abnormal movements and various paroxysmal non-epileptic motor disorders [179]. In *SLC2A1* mutation carriers, PED can occur with or without other manifestations of GLUT1-DS [2,57,113,181,182,183,184] and the age at onset varies from 1 to 50 years [1]. Beside PED, the range of PMD due to *SLC2A1* mutations is broad, including PKD, PNKD, EA, HA, abnormal eye–head movements, writer’s cramp, and dystonic tremor [35,48,112,113,116,117,182,183,185,186,187,188]. 

Most cases are caused by de novo heterozygous mutations, only 10% of the patients having an affected parent. Rarely, GLUT1-DS can be due to biallelic mutations, usually with asymptomatic heterozygous parents [189,190]. Small-scale mutations accounts for most of the cases, but intragenic and whole-gene deletions have been reported in a significant number of cases [191]. Milder forms are usually due to missense variants, while nonsense and splice site variants and insertions, deletions, intragenic and whole-gene deletions cause moderate to severe forms [179]. Exon 4, encoding for the fourth transmembrane domain of GLUT1, has been suggested as a vulnerable region of the protein [179]. Several mutational hotspots have been reported, with variants affecting the p.R126 and p.R333 residues frequently involved in PMD [58]. Hypoglycorrhachia (<60 mg/dl) and low cerebrospinal fluid (CSF)/blood glucose ratio (usually < 0.4, but can be higher in milder forms) are supporting findings. 3-O-methyl-D-glucose uptake assays are considered the gold standard to reliably assess GLUT1 residual activity [192], but are time- and cost-expensive. Assessment of GLUT1 expression on erythrocyte surface by flow cytometry has been proposed as an easier, alternative tool [193].

Disease manifestations are attributable to neuroglycopenia resulting from insufficient glucose transport across the BBB, causing brain energy failure. A 25%–35% reduction in GLUT1 activity is believed to underlie milder forms, more severe phenotypes resulting from 40%–75% reductions [190]. Paroxysmal events are probably due to the inability to meet brain glucose needs under circumstances of increased demand, and their higher frequency in infancy and childhood probably reflect the elevated cerebral metabolic rate for glucose during development [179]. Ketogenic diet (with different lipid/carbohydrate ratios) is considered the standard treatment, providing an alternative source of energy by switching brain metabolism from glucose to ketone bodies [194]. To overcome adherence issues (especially in adult patients), an alternative strategy using triheptanoin (an odd-chain synthetic triglyceride) has proven sustained efficacy on PMD in a group of patients [195,196,197].

#### 4.1.4. Pyruvate Dehydrogenase Complex (PDH) Deficiency (OMIM #300502, #608769, #608770) 

PDH deficiency presentation may range from early-infantile acute encephalopathy with lactic acidosis to chronic disorders, including Leigh syndrome and various combination of spasticity, ataxia, dystonia, peripheral neuropathy, optic atrophy, seizures, and intellectual disability [198,199]. A broad range of PMD are described in PDH deficiency, including episodic weakness [200], intermittent ataxia [201,202] and PxDs. PxDs may present as PED or as PNKD, sometimes with hemidystonic attacks. They may occur as isolated manifestation or, more frequently, within complex neurological phenotypes [59,60,198,203,204,205,206,207]. Typical hallmarks of PDH deficiency (such as elevated lactate levels or low lactate/pyruvate ratio in serum and CSF) or MRI abnormalities (such as bilateral pallidal hyperintensities or agenesis of the corpus callosum) may suggest the diagnosis, but their absence does not rule out PDH deficiency [59]. 

PDH complex is composed of three catalytic subunits: pyruvate dehydrogenase (E1, a heterotetramer of 2 E1α and 2 E1β subunits, respectively encoded by *PDHA1* and *PDHB1* genes), dihydrolipoamide acetyltransferase (E2, encoded by *DLAT*), and dihydrolipoamide dehydrogenase (E3, encoded by *DLD*), and by an additional component, the E3-binding protein (encoded by *PDHX*) [1,208]. The E3 subunit is common to three mitochondrial enzyme complexes (PDH, branched chain α-ketoacid dehydrogenase, α-ketoglutarate dehydrogenase), so *DLD* defects cause multiple biochemical defects [209]. PDH complex catalyzes the oxidative decarboxylation of pyruvate with the formation of acetyl-CoA, linking the glycolytic pathway to the Krebs cycle and playing a pivotal role in glucose metabolism in fed and fasting states (Figure 4) [208]. *PDHA1*, *DLAT*, and *PDHX1* defects have been linked to PxDs [1]. *PDHA1* defects are inherited with an X-linked pattern and represent the main cause of PDH deficiency, while *DLAT* and *PDHX1* defect are autosomal recessive [199]. Regardless of the genetic defect, no clear correlation between residual PDH activity and phenotype exists [199]. For *PDHA1*, small-scale mutations, intragenic deletions and whole-gene rearrangements have been reported [205,210,211,212,213]. Overall, missense are more common than frameshift mutations, but frameshift mutations are more common in symptomatic females [199]. Intragenic *PDHX* deletions have also been reported [210]. 

PDH deficiency is a potentially treatable disorder, responding to ketogenic diet [199]. Supplementation with thiamine (the cofactor of the dehydrogenase) may be effective in patients harboring *PDHA1* mutations affecting the E1α-thiamine binding site [59,199].

#### 4.1.5. ECSH1 and HIBCH (OMIM #602292 and #610690) 

PxDs have been associated with the deficiency of two mitochondrial enzymes involved in branched-chain amino acids metabolism: short-chain enoyl-CoA hydratase and 3-hydroxyisobutryl-CoA hydrolase, respectively encoded by *ECSH1* and *HIBCH* genes. 

*ECSH1* mutations mostly cause autosomal recessive early-onset Leigh syndrome but have been recently reported in patients showing PED. All patient reported so far with the milder PED phenotype were compound heterozygous for the pathogenic c.518C > T variant and a second pathogenic variant [62,63,214]. In addition, paroxysmal opisthotonus attacks with no clear trigger have been reported in patients with more severe *ECSH1*-related syndromes [215]. Patients with PED presentation may lack the abnormalities in the organic urine acids excretion commonly reported in more severe forms [62], but bilateral pallidal hyperintensities seem to be a constant finding [62,63,214]. A possible benefit with a mitochondrial vitamin cocktail has been reported [62].

*HIBCH* mutations are described as a rare cause of early-infantile mitochondrial encephalopathy with progressive dystonia [216]. A milder phenotype with PED, hyperCKemia, hyperammonemia, raised serum lactate during exercise and pallidal hyperintensities (without interictal neurological deficits, plasma acylcarnitines alteration or organic acid excretion abnormalities) has been reported in a single patient harbouring biallelic *HIBCH* mutations (p.H343D and p.V128D), showing good response to low-valine diet [64]. Valine dietary restriction is a potential treatment strategy for both *ECSH1* and *HIBCH* defects, preventing accumulation of toxic valine catabolites (Figure 4). Ancillary treatments could imply the supplementation of carnitine (to promote the excretion of 3-hydroxyisobutyryl-CoA as 3-hydroxyisobutyryl-carnitine, reducing the substrate for production of the toxic methacrylyl-CoA) and administration of detoxifying drugs, such as cysteamine and N-acetylcysteine, for replenishing intramitochondrial glutathione (consumed by increased methacrylyl-CoA) [216].

#### 4.1.6. ATP1A3 (OMIM #182350) 

Mutations in *ATP1A3* gene are responsible for a continuum spectrum of disorders encompassing different entities, such as early-infantile epileptic encephalopathy (EIEE), AHC, cerebellar ataxia with pes cavus, optic neuropathy and sensorineural hearing loss (CAPOS), recurrent encephalopathy with cerebellar ataxia (RECA), and rapid-onset dystonia-parkisonism (RDP) [107,217,218,219]. *ATP1A3*-manifestations exhibit an age-dependent pattern of emergence and progression, with a high prevalence of paroxysmal or episodic motor symptoms [220,221]. 

In newborns and young infants, paroxysmal abnormal ocular movement and dyskinetic bouts, together with dysautonomic episodes, are the distinguishing features of the most severe end of the spectrum, namely EIEE and severe AHC forms [218,219,222,223,224]. 

HA appear within the first 18 months and are the hallmark of AHC. Attacks highly vary in duration and frequency (even in the same patient), typically recognize emotional or environmental triggers (exercise, exposure to light, sounds or hot water, specific foods) and are relieved by sleep [114,225,226]. 

From early childhood, paroxysmal dystonia is almost a constant finding in AHC, but is also reported in RDP and intermediate phenotypes, or rarely as an isolated symptom [227,228,229]. Dystonic attacks involve one limb or one hemibody but can be generalized [180,181]. Distinguishing features of paroxysmal dystonia in *ATP1A3* include the preferential involvement of upper limbs, the unilateral or strikingly asymmetrical distribution, the long duration (for hours to days) and the frequent painful character [228].

In children, the paroxysmal onset of ataxia has been reported, mainly in the context of CAPOS and RECA phenotypes [217,230]. Both forms present in late infancy or childhood with relapsing episodes of cerebellar ataxia, triggered by fever or other stressors and variably associated with encephalopathic features, weakness, bulbar symptoms or hyperkinetic movements [107,217,221,225,231,232,233]. A recovery phase usually follows each episode, mimicking an EA course [107]. Nevertheless, recovery is slow and often incomplete (especially after recurrent decompensation episodes), leading to stepwise neurological deterioration [217]. The co-occurrence of peripheral, auditory and/or optic neuropathy distinguishes CAPOS from RECA [217]. 

*ATP1A3* encodes for the α_3_ subunit of the Na^+^/K^+^-ATPase, a ubiquitous, electrogenic transmembrane protein located on the cytosolic side of the outer plasma membrane. The α subunit is the catalytic component of the pump, and 4 isoforms exist, encoded by different genes and expressed in a tissue- and cell-specific manner [234]. The α_3_ subunit is selectively expressed in neurons, especially in GABAergic neurons of basal ganglia and cerebellum, whose membrane potential is largely dependent on the function of the pump [235]. Disease-causing mutations are sparse thorough the whole coding sequence, especially affecting the sites for ion binding and transport or enzyme phosphorylation. The CAPOS and RECA are genetically homogenous, due respectively to the variant p.Glu818Lys and to mutations involving the p.R756 residue [107,217]. RDP- and AHC-causing variants are largely distinct, but several pathogenic variants have been reported to cause different phenotypes, even in the same family [226,227,236,237]. For the most frequent AHC-causing variants, a gradient of symptoms severity has emerged (p.E815K > p.D801N > p.G947R). Despite the recent advances, the molecular underpinnings of the large phenotypic variability remain elusive [2,114]. 

#### 4.1.7. ADCY5 (OMIM #600293) 

Mutations in *ADCY5* result in a broad spectrum of paroxysmal and non-paroxysmal movement disorders [238], with variable degrees of severity. Axial hypotonia, with impact on motor development, is a frequent early finding, appearing in infancy [69]. The non-paroxysmal motor phenotype usually consist of mixed hyperkinetic movements, featuring variable combinations of chorea, athetosis, dystonia, and myoclonus [238]. Perioral and periorbital dyskinesias, resembling facial myokymia, are considered a typical finding [238]. The disease course is stable or slowly progressive [2]. 

PxDs are a core feature of *ADCY5*-related disorder, albeit often not falling into classic PxDs categories. Nocturnal dyskinetic bouts are a hallmark of the disease, but PxDs occur also during wakefulness and resemble PKD, PNKD, or PED [69]. The attacks often feature trunk dystonia and limb ballistic movements, lasting for minutes to hours with discrete onset and cessation [69,238]. They are often (albeit not always) painful, an element of distinction with most PxDs [1]. Nocturnal bouts occur both in NREM and REM sleep, determining prolonged awakenings [68,239]. Frequency and severity of PxDs often fluctuate over weeks to months with no apparent reason [238]. Rarely, the occurrence of HA has been reported, in the context of complex neurological picture including dysarthria, dystonia and chorea [110]. 

Although some discrete phenotypes are emerging [238], the clue for clinical suspicion of *ADCY5*-related disorder is the combination of pleiotropic nocturnal and diurnal PxDs with baseline mixed hyperkinetic movements [2]. Variable effects on dyskinesias have been reported with acetazolamide, benzodiazepines, tetrabenazine, propranolol, and several AEDs [110,238,240]. In addition, a benefit with caffeine assumption [241] and improvement after DBS have been reported [242,243].

*ADCY5*-related disorder arises from both de novo and autosomal dominantly inherited heterozygous mutations [244]. Nevertheless, few autosomal recessive cases, presenting with early onset generalized dystonia and myoclonus, have been described [245,246]. The arginine 418 residue seems to be a mutational hotspot, with the recurrent p.(R418W) variant causing a more severe phenotype compared with the p.(R418Q) and the p.(R418G) variants [244]. Somatic mosaicism is responsible for a relatively high proportion of apparently de novo cases, usually causing milder phenotypes [238]. *ADCY5* encodes for adenylate cyclase type 5 (AC5), a membrane-bound enzyme highly expressed in nucleus accumbens and striatum, where it catalyzes the conversion of adenosine triphosphate (ATP) into cyclic adenosine-3’,5’-monophosphate (cAMP), a second messenger for many signaling pathways [238]. AC5 interacts with multiple G-protein coupled receptors (GPCR), including adenosine A_2A_ and D_1_- and D_2_- dopamine receptors (Figure 2) [247]. A putative pathophysiological mechanism implies an abnormal increase of cAMP production, through a GOF mutational effect [248,249]. The involvement of striatum and nucleus accumbens respectively in motor control and sleep–wake cycle arousal, together with the stress-sensitive release of dopamine in these structures, may provide a functional- and anatomical basis to the sleep-related and stress-sensitive hyperkinetic phenotype [238]. In addition, A_2A_-adenosine receptors activate AC5. Caffeine assumption, through A_2A_-receptor antagonism, may be beneficial on dyskinesias [241].

However, the overproduction of cAMP by *ADCY5*-mutated cells has never been demonstrated in neural cell types. In addition, albeit heterozygous *ADCY5*-knock-out mice show a hypokinetic phenotype [247,250], human carriers of predicted LOF variants show a hyperkinetic phenotype [241,251]. The biological mechanisms of clinical heterogeneity and treatment response are still unclear.

#### 4.1.8. TBC1D24 (OMIM #613577) 

Homozygous or compound heterozygous TBC1D24 mutations cause a broad spectrum of neurological disorders, including several epileptic disorders, DOORS (deafness, onychodystrophy, osteodystrophy and mental retardation) syndrome or syndromic and non-syndromic deafness [252]. The occurrence of PED has been reported in association with epilepsy and/or interictal neurological abnormalities in several patients [253,254]. Attacks, triggered by physical exertion, combine trunk, limb, and facial dystonia [253], or may have focal distribution mimicking writer’s cramp [255]. Paroxysmal episodes start in infancy or childhood, but the exercise-induced character is uncertain at first episodes [253]. In a single patient, infantile-onset paroxysmal episodes of face myoclonus and limb tremors induced by fever or fatigue, later evolving into EA episodes, have been described [54]. As a result, the occurrence of PED or atypical PNKD in patients with early-onset complex neurological phenotypes encompassing epilepsy and/or ongoing abnormal movements should raise the suspicion of TBC1D24-related disorders. 

*TBC1D24* encodes for a presynaptic protein involved in vesicle trafficking (Figure 2). It contains both a Tre2/Bub2/Cdc16 (TBC) and a TBC/Lysin Motif Domain/Catalytic (TLDc) domain. The former interacts with Rab-GTPases, and docks to membrane phosphoinositides, regulating endo- and exocytosis [256]. The TLDc domain has been proposed to act as a sensor of oxidative stress at the synapse and regulate *TBC1D24*-dependent vesicle trafficking [254]. It has been suggested that the PED may occur in association with the presence of one missense mutation in TDLc domain (involving residues G501, G511, and A500) [254]. However, patients with PMD not harboring TDLc mutations have been reported [54,253], and phenotype–genotype correlations need to be exhaustively elucidated.

#### 4.1.9. SLC16A2 (OMIM #300095) 

*SLC16A2* encodes for the monocarboxylate transporter type 8 (MCT8), which facilitates transport of free triiodothyronine (fT_3_) at the BBB. MCT8 deficiency causes a severe, progressive disease known as Allan–Herndon–Dudley syndrome, presenting with developmental delay, hypotonia, microcephaly, ataxia, and progressive spasticity [257]. Some patients may develop a characteristic type of PKD triggered by passive movements, consisting in brief dystonic attacks lasting few seconds or minutes. More rarely, attacks may be precipitated by excitement or crying [36,258]. Raised fT3 plasma levels are the diagnostic clue of MCT8 deficiency. Brain MRI in infancy and early childhood frequently shows severely delayed myelination, which may subsequently improve over time [257]. The disorder is X-linked, and about 15% of the cases are due to (multi)exon or whole-gene deletions, often involving exon 1 [257,259]. Treatment with a T_3_ analog, TRIAC (acid 3,3′5-triiodothyroacetique), has proved to normalize fT_3_ blood levels and to reduce the thyrotoxic effects on peripheral tissues [260], but the effects on neurodevelopment are under investigation.

#### 4.1.10. Other Genetic Causes of PMD 

Beside the most common genes, several other genetic causes for PxDs have been reported. A single, heterozygous pathogenic mutation in the *SCN8A* (OMIM #600702) gene was first reported in 3 families presenting with BFIS or ICCA, mimicking the *PRRT2*-related phenotype [33]. All patients were found to carry the same, heterozygous missense mutation p.(E1483K). However, this association was questioned because of the evidence of an ictal cortical EEG correlate during a dyskinetic spell in one patient, suggesting an epileptic nature of the attack [1]. *SCN8A* encodes for the voltage-gated sodium channel subunit α type 8 (Nav1.6), highly expressed in cortex, basal ganglia and cerebellum [34]. It localizes predominantly on the axon initial segment, where it regulates action potential initiation and propagation [261]. A subsequent study investigating a large cohort of *PRRT2*-negative PKD patients identified another missense, likely pathogenic variant (p.A1214T) in a single patient with PKD without epilepsy [34], suggesting to consider this gene in the differential diagnosis of PxDs [1]. 

Mutations in the *KCNMA1* gene (OMIM #600150) have been reported to cause both PNKD and PKD [34,262,263], associated with epilepsy or developmental delay. *KCNMA1* encodes for the α subunit of the “Big K^+^” (BK) large conductance calcium and voltage-activated K^+^ channel. BK channels are widely expressed in CNS, where their activation reduces neuronal firing rates and presynaptic neurotransmitter release [264]. 

Anecdotal reports have linked PKD to mutations in *KCNA1* (see Section 3.2.1), as well as to autosomal recessive spastic ataxia of Charlevoix-Saguenay, Spinocerebellar ataxia type 8 and *CLCN2*-leukoencephalopathy [7,8,35,265]. PKD have also been linked to mutations in *DPEDC5*, encoding for the Dishevelled, Egl-10 and Pleckstrin domain-containing protein 5 (a subunit of a GTPase-activating protein complex involved in regulation of the mTOR pathway) [34,266] and *CHRNA4* (in association with epilepsy with febrile seizures) [38], encoding for the α4 subunit of the nicotinic acetylcholine receptor. Both these genes are known to cause nocturnal frontal lobe epilepsy, and the epileptic or dystonic nature of paroxysmal attacks remain to be elucidated [1].

In rare cases, PED can be the first manifestation of *PARKIN* (OMIM # 600116) or *GCH1* (OMIM #600225) mutations, usually causing juvenile parkinsonism and DOPA-responsive dystonia, respectively [65,66]. These reports highlight as PED can result from both dopamine and energy deficiency. Similarly, paroxysmal stiffening is considered a clue finding in infants with sepiapterin reductase deficiency, another disorder of dopamine synthesis [267]. 

Moreover, PxDs (both PKD and PNKD) are reported as a possible feature of idiopathic basal ganglia calcification (Fahr disease) [37,42,268,269,270].

In addition, PxDs have been described in patients with *PDEA10* LOF mutations, usually causing non-paroxysmal, early-onset chorea [271]. *PDEA10* encodes for the Phosphodiesterase 10A, an enzyme highly abundant in striatal medium spiny neurons, where it catalyzes the hydrolysis of cAMP and cGMP and modulates GPCR-signaling [244]. 

PxDs have been reported in a number of neurometabolic diseases, including Wilson, Lesch-Nyan and maple syrup urine disease (MSUD) and GABA transaminase deficiency [272,273,274,275]. 

Finally, hyperekplexia-like episodes have been reported in *SCN8A*- and *KAT6*-related encephalopathies [106,276].

### 4.2. Genes in EAs 

#### 4.2.1. KCNA1 (OMIM #176260)

Voltage-gated potassium channels (Kv) are the largest family of genes in the K^+^ channel family, with 40 genes representing 12 protein subfamilies K_v_1-K_v_-12. The K_v_1 subfamily plays a pivotal role in the onset and modulation of action potentials. The Kv1 family channels are broadly expressed. Kv1.1, Kv1.2, and Kv1.4 are the most highly expressed subunits in the CNS. The *KCN1A* gene, located at 12p13, codes for the Kv1.1, which is strongly expressed in the "basket" cells and in the interneurons, that form GABAergic synapses on Purkinje cells. It plays a key role in controlling neuronal excitability through membrane repolarization after an action potential. Several mutations in *KCNA1* causing EA1 have been reported, most of them being missense variants causing loss of channel function. Genotype–phenotype correlations are weak, and clinical variability has been reported also in twins carrying the same mutation (see Section 3.2.1 for description of EA1 and clinical variability), thus suggesting a possible contribution of non-genetic factors to phenotypic variability [277]. Besides EA1, heterozygous *KCNA1* variants have been found in progressive cerebellar ataxia [278] also with cognitive delay [279], epileptic encephalopathy [280], epilepsy and myokymia without EA [281] and PKD [35]. Finally, the first report of homozygous p.(V368L) mutations resulting in complete loss of channel activity has been recently shown to result in epileptic dyskinetic encephalopathy [282].

#### 4.2.2. CACNA1A (OMIM #601011) 

The voltage-gated calcium channels (VGCC) are multi-subunit complexes which regulate the intracellular concentration of calcium ions Ca^2+^. After entering the cell, Ca^2+^ activates specific calcium receptor proteins, (e.g., calmodulin, troponin C, or calcium-activated calcium, potassium, and chloride channels). In neurons, they play a pivotal role in neurotransmitter release. The first classification of VGCC was based on electrophysiological properties, specifically according to the membrane potential at which they are activated: They were classified in low-voltage-activated (i.e., at a membrane voltage positive to −70 mV), also known as T-type channels, and high-voltage-activated (i.e., at membrane voltages positive to −20 mV) [283]. The first known high-voltage-activated channel family, named L-type channels, was found in muscles (smooth, skeletal, and cardiac muscle) and neurons. Successively, other neuronal calcium channels were discovered and classified in N-type (N for Neuronal) and P/Q type (P for Purkinje cells). The second classification was developed by cloning of cDNA encoding channel types. It was proven that calcium channels consist of the principal α1 subunit, that is responsible for electrophysiological and pharmacological properties, and several auxiliary subunits β, α2δ, and γ, which have regulatory functions and each of them has several subtypes. There are at least 6 classes of α-1 subunits: α-1A, B, C, D, E, and S, which are encoded by 6 different genes [283]. The α-1A subunit is encoded by *CACNA1A* gene. It is located at 19p13.13 and contains 47 exons. The wide spectrum of neurological disorders associated with heterozygous *CACNA1A* ranges from progressive or non-progressive cerebellar syndrome to paroxysmal epileptic and non-epileptic phenotypes and includes familial hemiplegic migraine type 1 (FHM1) [284], EIEE type 42 [285], congenital-ataxia [286], EA2 [135,136], and SCA6 [287]. Additionally, a possible involvement in pathogenesis of autism has been postulated [288]. In general, phenotypes are frequently overlapping and may co-exist. Biallelic mutations have also been reported in a severe encephalopathic phenotype with progressive cerebro-cerebellar atrophy [289]. Different types of mutations are known, including point mutations, deletions, large gene rearrangements, and small expansion of CAG repeats. Both LOF and GOF mechanisms have been proven by functional studies. LOF has been proposed as mechanism mainly underlying EA2 [135,290], GOF is prevalent in FHM1, both LOF and GOF are observed in epileptic phenotypes [291]. SCA6 is caused by CAG expansion [287]; the normal number of CAG repeats ranges up to 18, while with SCA6 cases have 20–33 CAG repeats. With regard to EA2, it is worth note that many variants have been reported, including small intragenic deletions or duplications and point mutations (e.g., missense, nonsense, splice-site variants). However, phenotype did not differ significantly between cases harboring rearrangements and cases with point mutations. Specific *CACNA1A* pathogenic variants do not strictly predict the EA2 phenotype [290]. Estimated penetrance is of 80%–90%. Three pathogenic variants (p.R1281*, p.F1406C, p.R1549*) have been associated with fluctuating weakness manifesting as a myasthenic syndrome in patients affected by EA2 [292].

#### 4.2.3. CACNB4 (OMIM #601949) 

The β4 subunit of the P/Q type voltage-gated calcium channel CaV2.1 is encoded by the *CACNB4* (*calcium channel, voltage-dependent, beta-4 subunit*) located at 2q22-23. *CACNB4* undergoes alternative splicing and the resulting variants (β_4a_, β_4b_, β_4c_, β_4e_) display some distinct subcellular localizations and functions. Functional interaction between the α1 and the beta subunits modulate calcium currents [283]. The β4 subunit is highly expressed in the cerebellum [283]. Burgess and colleagues firstly demonstrated that a null mutation in the β4 subunit in the mutant “lethargic” mouse caused an autosomal recessive neurological disorder characterized by severe ataxia, focal motor abnormalities and absence seizures [293]. In humans, heterozygous variants in *CACNB4* have been discovered in different phenotypes: The p.(R482*) nonsense mutation has been found in a case of juvenile myoclonic epilepsy [144]; the p.(C104F) nonsense variant has been detected in patients with idiopathic generalized epilepsy and EA [144,147]. Recently, the homozygous p.(L126P) variant has been discovered in two brothers affected by a neurodevelopmental disorder with severe intellectual disability, epilepsy, dystonic, and athetoid movement disorders [294].

#### 4.2.4. SLC1A3 (OMIM #600111) 

*SLC1A3* on chromosome 5p13.2 encodes excitatory amino acid transporter 1 (EAAT1), a glial high affinity glutamate transporter. EAAT1 is expressed in the brainstem and cerebellum and it regulates the glutamate-mediated signal by the clearance of glutamate after synaptic release. Altered EAAT1 causes excessive extracellular accumulation of glutamate and neurotoxic insults. Currently, only missense mutations have been discovered (p.M128R, p.C186S, p.P290R, p.T318A, p.A329T, p.V393I, p.R399Q) [146,147,148,149,150,151]. In a case series of Korean EAs patients, one individuals harbored both *SLC1A3* p.(A329T) and *CACNA1A* p.(E1294del) variants, suggesting that *SLC1A3* as a possible genetic modifier for EA2 [151].

#### 4.2.5. UBR4 (OMIM #609890) 

The *UBR4* (Ubiquitin protein ligase E3 component N-recognin 4) gene is located at 1p36.13 and encodes a ubiquitin ligase protein that is known to interact in the nucleus with the retinoblastoma-associated protein and in the cytoplasm with Calmodulin (a Ca2+ protein involved in cellular calcium signaling), with ITPR1 (an intracellular receptor for inositol 1,4,5-trisphosphate which is highly expressed in the cerebellum and is involved in the regulation of Ca^2+^ homeostasis) and with smooth endoplasmic reticulum (a major intracellular calcium storage). UBR4 is hypothesized to act as a sensor of Ca2+, which is released through ITPR1 [152]. The fact that mutations in *ITPR1* can result in adult-onset ataxia (SCA15) or non-syndromic (SCA29) or syndromic congenital cerebellar ataxia (Gillespie syndrome) reinforces the role of *URB4* in the pathogenesis of cerebellar dysfunction [295,296,297]. Variants in *URB4* has been found both isolated and coupled with *CACNA1A* variants, thus suggesting also a role as genetic modifier [151]. Currently, five different missense variants (i.e., p.R5091H, p.T4877C, p.R4111H, p.A5042V, and p.A2581V) have been reported [151,152]. 

#### 4.2.6. Metabolic Disorders with EAs, Other Genes in Unclassified EAs, or Neurogenetic Diseases with EAs

This section covers other genetic causes of EAs, including some metabolic diseases which may rarely manifest with intermittent episodes of acute ataxia and neurogenetic diseases in which EA represents an allelic disorder. Only few sporadic or familial cases have been reported for each condition. 

##### Metabolic Disorders with EA

*MSUD* is a rare aminoacidopathy, caused by branched-chain 2-keto acid dehydrogenase (*BCKD*) complex deficiency, with subsequent accumulation of branched-chain amino acids leucine, isoleucine and valine. Most (80%) patients develop the classic form of MSUD with onset in the first days of life of maple syrup odor in cerumen and urine, ketonuria, poor feeding, and irritability evolving into encephalopathy with lethargy, intermittent apnea, opisthotonus, stereotyped movements. Some (20%) patients develop intermediate or intermittent MSUD types. Intermittent MSUD patients usually have normal psychomotor development but during intercurrent febrile illnesses and catabolism develop metabolic crises whose symptoms may vary from encephalopathy to EA [298]. Brain MRI may reveal T2 hyperintensities in cerebellar deep white matter and nuclei, dorsal pons, globi pallidi, thalami and cerebral white matter; diffusion-weighted images and apparent diffusion coefficient show acute diffusion restriction in myelinated areas as also seen in nonketotic hyperglycinemia and Canavan disease [299]. 

*Citrullinemia* is a metabolic disease belonging to the group of the urea cycle disorders. Patients with citrullinemia usually present in the neonatal period with life-threatening hyperammonemia and progressive encephalopathy with poor feeding, lethargy, vomiting, and hypothermia. Rare cases harboring mild childhood or adult onset form have been described. Recently, an 11-year-old boy with late-onset citrullinemia manifesting as episodes of ataxia, significant citrullinemia with mild hyperammonemia has been reported [300]. Episodes were triggered by minor febrile illnesses and lasted up to 7 days. The patient had normal interictal neurological examination, mild cerebellar atrophy on brain MRI, and harbor a compound heterozygous mutation in the argininosuccinate synthetase gene 1 (*ASS1*) gene [300]. 

The m.8993T > C mutation in the mtDNA ATP synthase subunit 6 gene (*MTATP6*) has been described in two main mitochondrial disorders, namely Leigh syndrome and neuropathy, ataxia and retinitis pigmentosa (*NARP*). A strong relationship exists between the level of mtDNA mutation and the disease severity: In particular, individuals harboring the m.8993T > C mutation manifest disease symptoms at heteroplasmy levels of more than 90% [301]. Craig and colleagues in 2007 screened for the m.8993T > C *MTATP6* mutation a large sample of patients with unexplained ataxia (total number 308) or Charcot-Marie-Tooth (n. 96) and EAs (n. 191) and identified only a single family in which one adult subject had EA and transient hemiparesis [302].

Leukoencephalopathy with brainstem and spinal cord involvement and lactate elevation (LBSL) is a recessive disorder caused by mutations in the *DARS2* gene, which encodes mitochondrial aspartyl-tRNA synthetase. The phenotypic spectrum of LBSL vary from infantile onset and rapidly fatal disease to adult onset slow and mild disease. However, the childhood presentation followed by slow neurological deterioration is the most common phenotype [9]. Van Der Knaap and colleagues defined the neuroradiological pattern of LBSL which include the involvement of cerebral and cerebellar white matter demyelinating leukodystrophy with relative savings of *U*-fibers, the pyramidal tracts of the medulla oblongata, and the dorsal column and the corticospinal tracts of the spinal cord [303]. Lactate peak is observed in MRI spectroscopy [303]. A single case of a 25-year-old woman, presenting with a 3-year history of paroxysmal short-lasting episodes of exercise-induced gait ataxia and areflexia of the upper and lower extremities, was found to harbor the c.1825C > T p.(R609W) homozygous mutation in *DARS2* [304]. Brain MRI was compatible with several major and supportive MRI criteria of LBSL. Acetazolamide was effective in achieving good control of attacks [304]. 

As previously stated (see Section 3.1.4), EA has been included among the milder forms of PDH deficiency presenting with PMD [201,202]. Debray and colleagues have reviewed these cases in 2008 [305]: 12 cases have been described, all being male, with a median age of 12 months at first ataxic event. Five out of 12 cases showed a late-onset progressive neurodegenerative course and many of them presented some signs of basal ganglia and brainstem alteration during the course of the disease. 

##### Other Genes in Unclassified EAs or Neurogenetic Diseases with EAs

*FGF14* gene (OMIM #601515) is located at 13q33.1 and encodes fibroblast growth factor 14. FGF14 belongs to a subclass of fibroblast growth factors that are highly expressed in the developing and adult central nervous system, especially in Purkinje cells. It is involved in both neuronal excitability—through regulation of the presynaptic Cav2.1 calcium channel and the axonal Nav1.2 and Nav1.6 voltage-gated sodium channels—and synaptic transmission from granule cells to Purkinje cells; it also plays a role in synaptic plasticity and neurogenesis in the hippocampus [306,307]. A role for FGF14 in axonal trafficking and synaptosomal function was suggested in 2002 by observation that Fgf14-deficient mice developed ataxia and a paroxysmal hyperkinetic movement disorder [308]. One year later, heterozygous mutations in *FGF14* were identified as causative of an autosomal dominant form of spinocerebellar ataxia, successively classified as SCA type 27 [309]. Less than 50 cases are currently described in the literature [310,311,312]. A recent review of published cases [310] concludes that early-onset tremor (mean age of 12.1 ± 10.5 years) is the typical manifestation at the onset of disease and it is followed by gait ataxia later in life (mean age of 23.7 ± 16.7 years) accompanied by limb ataxia, dysarthria, or nystagmus. Other features of SCA27 that may distinguish it from other SCAs are orofacial dyskinesias, psychiatric symptoms, cognitive impairment, and extrapyramidal features [310]. Congenital onset ataxia has been also reported [311]. Additionally, heterozygous variants in *FGF14* have been discovered in a total of 12 patients from 6 families with EA [151,313,314,315,316,317] and the phenotype of *FGF14*-related EA has been recently delineated: wide range of age at onset (from childhood to adulthood), fever as main triggering factor, variable duration (minutes to several days, thus potentially mimicking febrile cerebellitis) and frequency of attacks, and a variable association with additional findings at neurological examination such as nystagmus, postural upper limb tremor, and learning disabilities. Both point mutation and gene deletions have been recorded. Authors proposed to classify this form as *Episodic Ataxia type 9* (EA9). Interestingly, ataxia is not the only PMD encountered in EA9, since some cases may manifest PNKD [317]. A large family with EA9 also showed that some affected individuals may present with childhood onset nystagmus and/or postural tremor and/or learning disabilities without EA [317]. 

*Senataxin (SETX)* gene mutations are known to cause ataxia with oculomotor apraxia type 2 (an autosomal recessive cerebellar ataxia with onset between age three and 30 years after initial normal development, axonal sensorimotor neuropathy, oculomotor apraxia, cerebellar atrophy, and elevated serum concentration of α-fetoprotein) and juvenile amyotrophic lateral sclerosis type 4 (an autosomal dominant amyotrophic lateral sclerosis with mean age of onset at 17 years, slowly progressive distal amyotrophy, pyramidal signs, normal sensory examination, and lack of bulbar involvement). In a 4-year-old boy with normal development, unremarkable interictal neurological examination and EA, a heterozygous three-base pair deletion in *SETX* (c.135203911_135203913delTCA; p.D1024del) was detected by clinical exome sequencing. Unfortunately, segregation analysis on parents was not conducted to better asses the role of the variant [318]. Further studies are needed for understanding the role of *SETX* mutations in EA. The *CEP290* gene on 12q21.32 codes for a centrosomal protein involved in ciliary assembly and ciliary trafficking. Recessive mutations in CEP290 are known to cause Joubert syndrome type 5—a congenital ataxia due to a cerebellar malformation, manifesting with neonatal breathing abnormalities, hypotonia, ataxia, oculomotor apraxia associated with retinal and renal involvement—and the overlapping phenotype of Senior-Loken syndrome (retinitis pigmentosa and juvenile nephronophthisis) [319,320]. One 54-year-old Filipino man with a history of childhood onset intermittent diplopia, adult onset focal epilepsy and EA has been found by NGS Ataxia Panel to harbor the compound heterozygous mutations c.6798G > A p.(W2266*) and c.2174A > C p.(E725A) in *CEP290*. Episodes of ataxia started around the age of 50 years and were characterized by ataxia, fatigue, diplopia, and dysarthria; no triggering factors were identified. Mild cerebral and cerebellar atrophy is described on brain MRI [321]. 

The sodium leak channel nonselective protein (NALCN) is a voltage-independent, nonselective, non-inactivating cation channel permeable to Na^+^, K^+^, and Ca^2+^. It is responsible for the neuronal background sodium leak conductance and regulates resting membrane potential and neuronal excitability. It is part of a large ion channel complex, the "NALCN channelosome", consisting of multiple proteins including UNC80 and UNC79. Biallelic *NALCN* variants have been described in individuals with infantile hypotonia, severe psychomotor retardation, characteristic facies 1 and disordered respiratory rhythm with central apnea (IHPRF1) [322,323], while heterozygous de novo *NALCN* missense variants in the S5/S6 pore-forming segments lead to congenital contractures of the limbs and face, hypotonia, and global developmental delay (CLIFAHDD) [324]. Recently, *NALCN*-related phenotype has been widened to include adult-onset permanent cerebellar ataxia [325] and congenital ataxia with childhood-onset episodic ataxia exacerbations [326], both with cerebellar atrophy on brain MRI. In particular Aoyagi and colleagues described a young girl with congenital ataxia who also developed at age 3.5 years intermittent episodes of malaise, irritability, diaphoresis, vomiting and ataxia lasting 15–30 minutes, up to 5 times per week, triggered by fatigue, strong emotions, quick movements or car travel. The episodes were controlled by acetazolamide administration [326]. 

As described for *KCNA1*, the *KCNA2* gene encodes the voltage-gated α subunit Kv1.2, that is abundantly expressed in the central nervous system, especially in the Purkinje cells. Mice null for Kcna2 were found to have a shortened lifespan, seizures, and ataxia [327]. Heterozygous gain- or loss-of function variants in *KCNA2* have been linked to different phenotypes such as EIEE type 32 [328], spastic-ataxia (recurrent LOF c.881G > A p.(R294H) mutation) [329], early onset progressive myoclonus epilepsy [330] and EA with drug-responsive epilepsy [331]. Corbett et al. described a three-generation family in which seven affected individuals harbored the c.765_773del intragenic deletion, that caused dominant negative loss of channel function. Individuals presented with a variable phenotype ranging from isolated severe EIEE (one case) to epilepsy (six cases) possibly associated with delayed speech development (three cases) or mild intellectual disability (two cases) and with EA (five cases). EA started at a mean age of 8 years (range 5–12 years). Dysarthria was associated with ataxia, without ictal or interictal myokymia or nystagmus. Episodes were triggered by several factors (i.e., exercise, fatigue, illness, menstruation, startle, stress, and sudden movement) and may last from few seconds up to half a day. Acetazolamide was effective in reducing episodes in 2 cases [331]. 

*SCN2A* encodes the α subunit of the voltage gated neuronal sodium channel NaV1.2. Pathogenic variants in *SCN2A* have been reported in a wide spectrum of neurodevelopmental disorders including developmental and epileptic encephalopathies (EIEE11), benign familial neonatal-infantile seizures (BFIS3), autism spectrum disorder and intellectual disability with and without seizures, and EA. To date, more than 300 patients with *SCN2A* variants have been published, the majority presenting with epilepsy [332]. EA represents one of the less common phenotypes in the *SCN2A*-related disorders. Recently, Schwarz and colleagues defined the main features of *SCN2A*-related EA in a total of 21 cases, including 12 previously and 9 newly reported cases [333]. The authors found that most of patients (86%) presented with epilepsy, more often in the first three months of life (67%), while EA begun between 10 months and 14 years of age. Frequency of episodes may range from daily to yearly and their duration may vary from seconds or few minutes to several hours or days. Some patients may report triggering factors such as minor head trauma, sleep deprivation, menstruation cycle, sleep deprivation, and physical stress. Response to acetazolamide is variable; AEDs do not seem to reduce the frequency of ataxic attacks. Cognitive development is preserved or only mildly affected in the majority of cases (81%). Point mutations have been found in all *SCN2A*-related EA; the p.(A263V) mutations and variants affecting the S4 segment and its cytoplasmic loop within the domain IV represent mutational hotspots [333].

The spectrum of the PRRT2-related paroxysmal disorders includes PKD, BFIE, ICCA, PHD, HM, and also EA (see Section 3.1.1) [58,334]. In a study screening for *PRRT2* mutations in sample of 182 EA patients, Gardiner and colleagues found only one case manifesting EA and HM and harboring the common c.649dup p.(R217Profs*8) mutation in a heterozygous state [58]. Episodes of ataxia started at age 18 years and were associated to unilateral headaches and hemiplegia. Cerebellar ataxia on neurological examination and normal brain imaging were described. This indicates both that *PRRT2* mutations are rarely associated with a (complex) EA phenotype and that the genotype–phenotype correlations for this gene are elusive. Labate et al. described a consanguineous Italian family in which four affected members carrying the c.649dup p.(R217Profs*8) mutation in a heterozygous state showed a mild phenotype with only BFIS, while two members with the homozygous mutation presented a more severe phenotype characterized by BFIS, PKD, absences, intellectual disability and, in one case, EA [166]. Attacks of ataxia in this case appeared at age of 5 years, after onset of BFIS and PKD, and were characterized by gait unsteadiness and blurred vision with preserved consciousness lasting from 60 minutes up to 24–72 hours. No triggering factors were identified; attacks disappeared after acetazolamide administration at low dose (250 mg/day). Brain MRI was unrevealing. The authors suggested that additive effect of double dose of the genetic mutation [166]. Transient cerebellar diffusion restriction (e.g., brain MRI showing hyperintensity in diffusion-weighted images and decreased apparent diffusion coefficient) was detected in the acute phase of the first ataxic episode occurring in a *PRRT2*-c.649dup-mutated 21-year-old women. The episode started after swimming and lasted few less than four days [335]. 

Finally, episodes of paroxysmal ataxia have been described in the setting of GLUT1-deficiency syndrome and ATP1A3-related disorders. Please refer to Section 3.1.3 and Section 3.1.6 and for detailed discussion of these forms. Few other explorative genes (e.g., *TGM6*, *TTBK2*, *KCND3*) have been found in sporadically found by whole exome sequencing in single patients or families with EA and deserve further confirmation [151].

### 4.3. Secondary (Acquired) Causes of PMD and EAs

#### 4.3.1. Acquired PMD

In several cases, PxDs can be the consequence of acquired nervous system diseases, including immune-mediated, vascular, metabolic, or neoplastic processes [336]. In acquired PxDs, onset is usually in adulthood, and interictal neurological abnormalities are commonly found. Multiple sclerosis (MS) is probably the most common cause [41,337,338,339]. MS is an acquired demyelinating disease of the CNS in which the immune system attacks the protective sheath (myelin) that covers nerve fibers in the brain and spinal cord. Signs and symptoms of MS vary widely and depend on the localization and the amount of nerve damage. Paroxysmal episodes are attributed to the ephaptic activation of axons within partially demyelinated lesions [340]. In MS, PxDs can feature both PKD and PNKD and can occur as the presenting manifestation [41]. Attacks are usually painful (in contrast with most genetic PxDs, with some above mentioned notable exceptions) and underlie lesions in the internal capsule, basal ganglia, mesencephalon, or posterior periventricular white matter [337,338]. If new lesions are found, steroid treatment can be beneficial, but symptomatic treatment with oxcarbamazepine or acetazolamide is often needed [341].

The occurrence of pain in MS-related PxDs raises the issue of their distinction from tonic spasms [341]. Tonic spasms consist of brief (30 s–5 min), frequent attacks of stereotyped and painful abnormal tonic postures (asymmetric legs and arms extension, opisthotonus and facial contractions), usually precipitated by hyperventilation, noise, tactile stimuli, or voluntary movement [342]. Many authors refer to tonic spasms as PxDs [342], others distinguish the two entities because of clinical peculiarities that do not make them fall into classical PxDs subtypes [343], and the appropriate terminology remains a subject of debate [341]. 

Of note, PxDs can be the feature of several treatable disorders, including autoimmune encephalitis [72,344,345], acute disseminated encephalomyelitis [346], antiphospholipid syndrome [347], Hashimoto encephalopathy [43], thyrotoxicosis and hyperthyroidism [44], hypo- and hyperglycemia [348,349], hypocalcemia [350,351,352], or vasculopathies [353].

#### 4.3.2. Acquired EAs

Few anecdotal descriptions of acquired disorders manifesting with recurrent and intermittent episodes of ataxia are known. These disorders mainly were represented by autoimmune diseases of the CNS developing in adult patients. Usually, attacks of ataxia are not the only disease manifestation as they are recorded in the context of more heterogeneous clinical pictures.

Intermittent acute episodes of ataxia and dysarthria have been firstly described in the setting of MS more than 60 years ago by Harry Lee Parker, who used the name of “periodic ataxia” [354]. Successively in 1959, Anderman and colleagues renamed these episodes “paroxysmal dysarthria and ataxia” [355]. Thereafter, several authors have used the term paroxysmal dysarthria-ataxia syndrome (PDA) to identify this neurological disorder, although it is not uniformly applied with reference only to symptomatic forms of EA [356]. Episodes of PDA, although rare, have been documented in few less than 50 cases in the setting of MS [357,358]. PDA in MS consist in brief (less than 1 minute) and transient attacks of dyssynergia involved all the body muscles, included those responsible for articulation of the speech. Differential diagnoses include focal seizures and vascular disorders; video-EEG recording and brain MRI can help in achieving the diagnosis. Available brain MRI data constantly reported brainstem lesions involving the red nucleus in the midbrain; cerebellar hemispheres may also be affected [357,358]. It is postulated that the midbrain lesions may lead to interruption of the cerebello–thalamo–cortical pathway with subsequent induction of parietal diaschisis manifesting with PDA [359]. Some authors proposed that adult onset and short episodes are the most specific features for distinguishing “symptomatic” (i.e., in the context of an acquired neurological disease) from primary (i.e., EAs) PDA [356]. Several AEDs (e.g., carbamazepine, lamotrigine, phenytoin, oxcarbazepine, levetiracetam) or acetazolamide have been used with variable results for treating episodes of PDA in MS [357,358]. Anecdotal case reports also described PDA in remitting-relapsing anti-GQ1b antibodies-negative *Bickerstaff’s-like brainstem encephalitis* [360], *stroke* [361], and *Bechet’s disease* [362]. An interesting case of an unexplained brainstem lesion causing paroxysmal ataxia, dysarthria, diplopia and hemifacial spasm (PADDHS syndrome) has been recently described [363]. In all cases the detectable lesions involved the brainstem and the red nuclei. 

*Paraneoplastic limbic encephalitis* is a rare disorder characterized by personality changes, seizures, memory loss, and psychiatric symptoms often associated with MRI changes (in about 50% of cases) of increased T2 signal in the mesial temporal structures, inflammatory CSF parameters, and positive antineuronal antibodies. Stereotyped episodes of short-lasting (i.e., minutes to few hours) gait imbalance, slurred speech, and limb dysmetria were reported in 25% of adult patients with *anti–contactin associated protein-like 2 (CASPR2) antibody-related autoimmune limbic encephalitis* presenting with amnesia and seizures [364,365]. Episodes developed during the course of encephalitis and disappeared after immunomodulating therapy, thus suggesting an immune origin of the ataxic manifestations. Interestingly, paroxysmal ataxia was not observed in the other two diseases associated with CASPR2 antibodies neuromyotonia and Morvan syndrome, a rare disorder characterized by peripheral nerve hyperexcitability, encephalopathy, dysautonomia and insomnia. A dysfunction of voltage-gated potassium K_V_1.1 channels (encoded by *KCNA1*) at the nodes of Ranvier caused by anti-CASPR2 antibody has been postulated as causative of neuromyotonia in anti-CASPR2 antibodies encephalitis [365]. A child with *anti-Hu-associated paraneoplastic limbic encephalitis* that presented with EA and behavioral changes evolving to intractable epilepsy has been reported [366].

## 5. Conclusions

**Figure 5:**
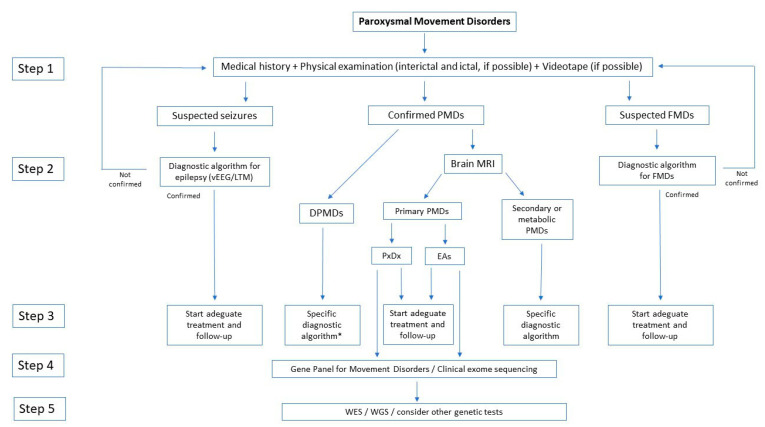
Operative flowchart for pediatric-onset PMDs. DPMDs: developmental PMDs; EAs episodic ataxias; FMDs: functional movement disorders; LTM: long term EEG monitoring; PxDx paroxysmal dyskinesias; vEEG: video electroencephalogram; WES: whole exome sequencing; WGS: whole genome sequencing. * Please refer to Table 3 for specific diagnostic algorithm for DPMs.

Although PMDs are rare diseases, taken all together they are quite often encountered in both adult and, especially, pediatric neurology practice. Some of these forms are even frequent (e.g., developmental PMDs) and their recognition can allow to shorten the diagnostic process and avoid inappropriate investigations (Figure 5 and Table 3). On the other hand, a correct identification of a rare PMDs can allow an adequate diagnostic and pharmacological approach. Recent advances in the genetics of the PMDs are constantly increasing our understanding of the pathophysiological bases and treatment options of these disorders. In consideration of both the high complexity of molecular mechanisms, the pleiotropic effect of many causative genes and the overlapping clinical features of different PMDs, a NGS approach by targeted panel for movement disorders, clinical or whole exome sequencing should be preferred—whenever possible, in consideration of costs and accessibility to NGS technologies—to single gene approach. Although it is likely that the diagnostic yield of targeted NGS panel for movement disorders depends on the selection of patients and the investigation strategies adopted, the overall rate of molecular diagnosis has been reported to range from 11% to 51% [366]. This allows to significantly reduce the number of patients who may need further molecular genetic investigations (e.g., whole exome or genome sequencing for undiscovered genes; search for triplet-repeat expansions or copy number variations) [366]. 

Although several aspects of PMDs have been clarified over the past decade, large areas of uncertainties and controversial issues persist. PMDs classification should be revised and implemented to include the expanding spectrum of different clinical manifestations, the increasing number of causative genes and the emerging genotype–phenotype correlations. In addition, further studies are needed to increase the rate of genetically solved cases and to improve treatment options, especially for drug-resistant and so far untreatable forms.

## Figures and Tables

**Table 1:** Main genetic causes of paroxysmal movement disorders. A question mark follows treatment options that: have been proposed basing on pathophysiological assumptions, are under investigation or have been shown to be beneficial only in single-case reports.

Gene	OMIM	Inheritance	Age at Onset	PMDs Subtype	Attack Duration	Isolated Versus Combined	Allelic Disorders	Other Possible Features	MRI	Treatment
**PRRT2**	**614386**	AD	<18 years	PKD	Very brief (<1 min)	I/C	BFIS, ICCA, FHM, EA		Normal	CBZ (PKD) ACZM (EA)
**PNKD**	**609023**	AD	<18 years	PNKD	Long (>1 h)	I	Migraine (rare), PKD			BDZ (Attacks relief)
**SLC2A1 (GLUT-1)**	**138140**	AD	Variable	PED, EA, HA, PEM	Intermediate (5–40 min)	I/C	Classic GLUT1-DS, HSP,	Anaemia, hypotonia, spasticity, seizures, Developmental delay/ID, dystonia, ataxia		Ketogenic diet, triheptanoin
**PDH complex (PDHA1/PDHX** **/DLAT)**	**300502/608769/608770**	AR	Infancy	PED/PNKD	Variable	I/C	Leigh syndrome	Developmantal delay/ID, Seizures, progressive dystonia	Pallidal hyperintensities, Callosal agenesis	Ketogenic diet
**ECHS1**	**602292**	AR	Infancy	PED	Variable	I/C		Leigh syndrome	From Pallidal hyperintensities to Leigh-like abnormalities	Valine-restricted diet? detoxifying drugs?
**HIBCH**	**610690**	AR	Infancy	PED	Variable	I/C	Leigh syndrome	ID, Seizures, progressive dystonia	From Pallidal hyperintensities to Leigh-like abnormalities	Valine-restricted diet? detoxifying drugs?
**ATP1A3**	**182350**	AD	Variable	PNKD ([hemi] dystonic attacks), HA, PEM	Variable	C	EIEE, AHC, CAPOS, RECA, RDP	Seizures, dysautonomic paroxysms, nonparoxysmal dystonia, ataxia, parkinsonism		Flunarizine (HA prophylaxis), BDZ (HA relief)
**ADCY5**	**600293**	AD	Variable	PKD/PNKD/PED/PND	Brief (minutes)	C	PNKD	Axial hypotonia, nonparoxysmal dystonia and chorea		Caffeine?
**TBC1D24**	**613577**	AR	Childhood	PED	Variable	C	Deafness, DOORS syndrome, Rolandic Epilepsy, EIEE16, Myoclonic epilepsy	Sizures, Developmental delay/ID, myoclonus, ataxia, extraneurological abnormalities		
**SLC16A2 (MCT8)**	**300095**	X linked	<1–2 months	PKD (triggered by passive movements)	Very brief (seconds to minutes)	C		Mental retardation		TRIAC?
**SCN8A**	**600702**	AD	Infancy	PKD	Brief	C	Epilepsy	Mental retardation		CBZ, oxcarbazepine
**KCNMA1**	**600150**	AD	Childhood	PNKD	Long (>1 h)	C		epilepsy, developmental delay, progressive HSP, ataxia		
**GCH1**	**600225**	AD	<18 years	PED	Variable	I/C	DRD	Non paroxysmal dystonia and parkinsonism		L-DOPA
**PDE10A**	**610652**	AR/AD	Childhood	PNKD	NR	C	Chorea without paroxysms	Dystonia, Parkinsonism, marked fluctuations	Striatal hyperintensities (in AD cases)	
**KCNA1**	**176260**	AD	Childhood (2–15)	EA1	Minutes	I	EIEE, PKD, EDE (AR)	interictal Myokymia; progressive ataxia (20%), epilepsy (10%)	Normal ore cerebellar atrophy (10%)	CBZ, PHT, ACZM
**CACNA1A**	**601011**	AD	Childhood (0–20)	EA2/PTU/BPT	Variable (minutes to days)	I/C	FHM1, SCA6, CA	progressive ataxia, Developmental delay	Normal or cerebellar atrophy	ACZM, 4-APD, LEV
**CACNB4**	**601949**	AD	Young-adult onset	EA5	several hours	I	JME, IGE, CND (AR)	Epilepsy, permanent ataxia	Normal	ACZM
**SLC1A3 (EAAT1)**	**600111**	AD	infancy or childhood (rarely adulthood)	EA6	several hours	I	Adult-onset progressive ataxia	Seizures (rare) HA	Nornmal; rarely cerebellar atrophy	ACZM
**UBR4**	**609890**	AD	around age 2 years	EA8	minutes to hours	I		nystagmus, myokymia, tremor		Clonazepam
**FGF14**	**601515**	AD	late-childhood to early adulthood	EA9	minutes	I/C	SCA27, CA	progressive ataxia, nystagmus, postural upper limb tremor, ID		
**BCKD Complex**	**608348/248611**	AR	Variable	EA/PNKD	Minutes to hours	C	Classic MSUD	developmental delay, progressive psychomotor retardation, seizures, ataxia,	T2 hypersignal in in the brainstem, globus pallidus, thalami, and dentate nuclei	BCAA restricted diet
**KCNA2**	**176262**	AD	Infancy or childhood	EA	Seconds to hours	C	EIEE32, SCA, PME	Epilepsy		ACZM (variable)
**SCN2A**	**182390**	AD	infancy or childhood	EA	minutes to days	C	EIEE11, BFIS3	Seizures +/− encephalopathy, developmental delay/ID	Normal or cerebellar atrophy	ACZM (variable)

4-APD: 4-amynopiridine; ACZM: Acetazolamide; AHC: Alternating hemiplegia of childhood; AR: Autosomic recessive; AD autosomic dominant; BDZ: benzodiazepines; BFIS: benign familial infantile seizures; BPTI: Benign paroxysmal torticollis of infancy; C. Combined; CA: congenital ataxia; CAPOS: cerebellar ataxia, pes cavus, optic atrophy, sensorineural hearing loss; CBZ: carbamazepine; CND: complex neurodevelopmental disorder; DOORS: deafness, onychodystrophy, osteodystrophy, mental retardation, and seizures; DRD: Dopa-Responsive Dystonia EA: episodic ataxia; EDE: epileptic dyskinetic encephalopathy; EIEE: Early infantile epileptic encephalopathy; FHM: familiar hemiplegic migraine; HA: hemiplegic attacks; I: Isolated; ID: Intellectual disability; JME: juvenile myoclonic epilepsy; LEV: levetiracetam; MSUD: maple syrup urine disease; PED: Paroxysmal exercise-induced dyskinesia; PEM: Paroxysmal eye movements; PHT: phenytoin; PKD: Paroxysmal kynesigenic dyskinesia; PNKD: Paroxysmal non-kynesigenic dyskinesia; PME: progressive myoclonic epilepsy; PTU: Paroxysmal tonic upgaze, RECA: recurrent encephalopathy with cerebellar ataxia; RDP: rapid onset dystonia-parkinsonism; SCA: spinocerebellar ataxia; VPA: Valproic Acid.

**Table 2:** Phenotypic classification of paroxysmal movement disorders.

PMD Type	Clinial Criteria	Main Genetic Causes	Rare Genetic Causes	Acquired Causes
**Paroxysmal Dyskinesias**				
**Paroxysmal kynesigenic dyskinesia**	Onset between 1–20y Triggered by sudden movements (kinesigenic) Duration of attacks < 1 min Good response to antiepileptic drugs	*PRRT2*	*SCN8A, SLC16A2, SLC2A1, DEPDC5, CLCN2, PNKD, KCNMA1, KCNA1, CHRNA4, SACS*, Wilson disease, Basal Ganglia Calcifications (*SLC20A2, PDGFB*)	Multiple sclerosis & other demyelinating diseases, Stroke (including vasculopathy), Autoimmune encephalopathies (antiNMDAr, CASPR2, VGKC, Hashimoto), Perinatal brain injury, CNS infections, Thyrotoxicosis, PSP, central pontine myelinolysis, Methylphenidate treatment, Functional Disorders
**Paroxysmal non-kynesigenic dyskinesia**	Onset of attack in infancy or early childhood Triggered by caffeine and alcohol intake Duration of attacks between 10 min–1 h (<4 h) Often responds to benzodiazepines	*PNKD*	*PRRT2, SLC2A1, ATP1A3, ADCY5, TBC1D24, KCNMA1, PDE10A, KCNA1*, BCKD complex, Neuroacanthocytosis, Lesch-Nyhan disease, GABA-Transaminase deficiency, Basal Ganglia Calcifications (*SLC20A2, PDGFB*)	Multiple sclerosis & other demyelinating diseases, Stroke (including vasculopathy), TIA, Hypo-/hyperglycemia, Systemic autoimmune disorders, Celiac disease, Perinatal brain injury, CNS infections, Parasagittal meningioma, Trauma, Functional Disorders
**Paroxysmal exercise-induced dyskinesia**	Onset of attack from childhood to adulthood Triggered by exercise (at least minutes of exercise) Duration of attacks between 5 min–30 min Treatment according with the underlying defect	*SLC2A1*	PDH complex (*PDHA1/PDHX/DLAT*), *HIBCH, ECSH1, GCH1, PARK2, ADCY5, TBC1D24*	
**Paroxysmal nocturnal dyskinesia**	Paroxysmal Bouts of non-epileptic dyskinesias occurring in sleep	*ADCY5*	*PRRT2*	Stroke, autoimmune encephalitis (NMDAr, IgLON5)
**Other Paroxysmal Movement Disorders**				
Benign Paroxysmal Toricollis of Infancy *	recurrent episodes of painless paroxysmal cervical dystonia (featuring latero-, retro- or torticollis) Onset 3–30 months Remission before 5y	*CACNA1A*		
**Hemiplegic attacks**	Onset within 18 months episodes of hemiplegia, alternating in laterality Possible quadriplegic attacks (in isolation or as generalization of HA) emotional or environmental trigger factors	*ATP1A3*	*ATP1A2, SLC2A1, SCN4A, ADCY5, TBC1D24, TANGO2*	
**Hyperkplexia**	excessive startling to unexpected, auditory or tactile stimuli	*GLRA1, GLRB, SLC6A5, ATAD1*	GM1 gangliosidosis, *SCN8A*, Pontocerebellar hypoplasia, Posterior fossa malformations	Perinatal injury, Postanoxic encephalopathy, trauma, Paraneoplastic, Multiple sclerosis, ALS, CNS Infections, Medulla compression, MSA
**Paroxysmal abnormal eye movements**				
**Paroxysmal Tonic Upgaze**	paroxysms of conjugate upward gaze down-beating saccades on attempts to downward gaze preserved horizontal eye movements unimpaired consciousness episode lasting hours (up to 48 h)	*CACNA1A* *	*GRID2*, Pelizaeus-Merzbacher Disease, Brain Malformations	Perinatal injury, hydrocephalus, brain tumors
**Oculogyric Crisis**	paroxysmal, tonic, conjugate, often upward ocular deviation lasting minutes to hours	Biogenic amine metabolism defects (GTPCH, TH, SPR, AADC, PTS, VMAT2, DAT)	*NOTCH2NLC* (NIID), *DCTN1* (Perry syndrome), *LYST* (Chediak-Higashi), *ATP13A2* (Kufor-Rakeb disease), *ATP1A3* (RDP) Rett syndrome	Dopamine-receptor blocking agents, encephalitis Lethargica, Anti-NMDAr encephalitis
**Saccadic eye-head gaze shifts**	brief episodes of eye–head movements	*SLC2A1* (GLUT1-DS)		
**Paroxysmal nystagmus**	paroxysmal episodes of monocular and binocular nystagmus, often disconjugate	*ATP1A3*		
**Episodic Ataxias**				
**Episodic ataxia**	Onset in childhood or adolescence (genetic EA) or adulthood (acquired EA) Attacks of ataxia, dysarthria, nystagmus lasting seconds (acquired EA), minutes (EA1) or hours to days (EA2)	*KCNA1, CACNA1A*	*CACNB4, CEP290, FGF14, KCNA2, NALCN, PRRT2, SLC1A3, SLC2A1, UBR4, SCN2A*, few neurometabolic and mithocondrial disorders	Multiple sclerosis & other demyelinating diseases, Stroke and Behcet’s Disease (brainstem and red nuclei involvement); Paraneoplastic limbic encephalitis with anti-CASPR2 or anti-Hu antibodies

* most cases are idiopathic and transient. AADC: Aromatic l-amino acid decarboxylase; ALS: Amyotrophic lateral sclerosis; BCKD: Branched-chain ketoacids dehydrogenase; BPTI: Benign paroxysmal torticollis of infancy; CASPR2: Contactin-associated protein-like 2; CNS: central nervous system. DAT: dopamine transporter. EA: Episodic Ataxia; GLUT1-DS: GLUT1 Deficiency syndrome; GTPCH: Guanosine Triphosphate cyclohydrolase I; MSA: multiple system atrophy; NIID: Neuronal intranuclear inclusion disease; NMDAr: N-methyl-D-aspartate receptor; PDH: Pyruvate dehydrogenase; PSP: Progressive supranuclear palsy; PTS: 6-Pyruvoyl Tetrahydrobiopterin Synthase; RDP: Rapid-Onset Dystonia-Parkinsonism; SR: sepiapterin reductase; TH: Tyrosine Hydroxylase; VGKC: voltage-gated potassium channel-complex; VMAT2: Vesicular monoamine transporter 2.

**Table 3:** Suggested investigations in Developmental Paroxysmal Movement Disorders (DPMDs).

Type of DPMDs	Suggested Investigations
**Benign neonatal sleep myoclonus**	No investigation required
**Paroxysmal Tonic Upgaze**	Perform EEG to differentiate from versive seizures If interictal neurological abnormalities are present (developmental delay, hypotonus, hypokinesia, dystonia, nystagmus), consider CSF sampling for neurotransmitter analysis (to differentiate from OGC) and MRI (to exclude brain abnormalities) Consider *CACNA1A* testing
**Benign Paroxysmal Toricollis of Infancy**	Consider *CACNA1A* testing
**Transient dystonia of infancy**	Perform MRI to exclude focal lesions
Benign polymorphous movement disorders of infancy *	No investigation usually required Consider EEG if manifestations are difficult to interpret

* including benign myoclonus of early infancy, shuddering attacks, shaking body attacks, non-epileptic head atonic attacks. CSF: cerebrospinal fluid, OGC: oculogyric crisis; EEG: electroencephalogram.
